# Associations of NMR metabolic biomarkers and arterial calcification: An observational and Mendelian randomization study within the BBMRI metabolomics consortium

**DOI:** 10.1016/j.athplu.2026.03.001

**Published:** 2026-03-10

**Authors:** Haojie Lu, Maxime M. Bos, Nicolien A. van Vliet, Mohsen Ghanbari, Marian Beekman, Luoshiyuan Zuo, Pooja Mandaviya, Dina Vojinovic, Carolina Medina-Gomez, Katerina Trajanoska, P. Eline Slagboom, BBMRI Metabolomics Consortium, Jeroen van der Grond, M. Arfan Ikram, Diana van Heemst, Daniel Bos, Maryam Kavousi

**Affiliations:** a Department of Epidemiology, Erasmus MC University Medical Center, Rotterdam, the Netherlands; b Department of Internal Medicine, Erasmus MC University Medical Center, Rotterdam, the Netherlands; c Section of Gerontology and Geriatrics, Department of Internal Medicine, Leiden University Medical Center, Leiden, the Netherlands; d Section of Molecular Epidemiology, Department of Biomedical Data Sciences, Leiden University Medical Center, Leiden, the Netherlands; e Department of Radiology and Nuclear Medicine, Erasmus MC University Medical Center, Rotterdam, the Netherlands; f Department of Radiology, Leiden University Medical Center, Leiden, the Netherlands

**Keywords:** NMR metabolic biomarkers, Arterial calcification, Observational associations, Sex-stratification, Mendelian randomization, Coronary heart disease

## Abstract

**Background and aim:**

The metabolic roles in arterial calcification remain largely unclear. We aimed to elucidate the associations between nuclear magnetic resonance (NMR) metabolic biomarkers with site- and sex-specific arterial calcification as well as their causal nature.

**Methods:**

This study included participants from the Rotterdam Study (RS, N = 1062), Leiden Longevity Study (LLS, N = 272), and UK Biobank (UKBB, N = 271,793). In RS and LLS, we applied linear regression (both overall and sex-stratified) to assess the association between NMR metabolic biomarkers and arterial calcification at coronary artery (CAC), aortic arch (AAC), and aortic valve (AVC). Two-sample Mendelian randomization (MR) was employed using genome-wide association study of NMR biomarkers (N = 115,082) and CAC (N = 26,909) to infer their genetic causality. Biomarkers with consistent observational and causal associations were further examined for associations with coronary heart disease (CHD) in UKBB.

**Results:**

From multiple-testing correction (P-value < 4.39 × 10^−4^), three fatty acids ratio biomarkers were associated with decreased CAC burden: Omega_6_pct (Beta: −0.10, SE: 0.03, P-value: 2.94 × 10^−4^), PUFA_by_MUFA (Beta: −0.10, SE: 0.03, P-value: 3.08 × 10^−4^), and PUFA_pct (Beta: −0.10, SE: 0.03, P-value: 3.33 × 10^−4^). Sex-stratification revealed glycoprotein acetyls (GlycA) associated with CAC in males only (Beta: 0.13, SE: 0.04, P-value: 3.10 × 10^−4^). MR identified 17 biomarkers causally associated with CAC, 14 of which were subsequently associated with CHD in UKBB.

**Conclusions:**

We identified NMR-based metabolic biomarkers, particularly fatty acid ratios, that were significantly and causally associated with CAC burden. GlycA was associated with CAC in males only. Replication in UKBB further underscores their clinical relevance with CHD.

## Highlights

• Three fatty acid ratio metabolic biomarkers: omega-6 fatty acids to total fatty acids, polyunsaturated fatty acids to monounsaturated fatty acids, and polyunsaturated fatty acids to total fatty acids, were found to be inversely associated with coronary artery calcification (CAC).
• Glycoprotein acetyls was associated with increased CAC only in males.
• Two-sample Mendelian randomization indicated genetic causal associations between 17 biomarkers and CAC.
• 14 biomarkers were subsequently validated as significantly associated with coronary heart disease in UK Biobank.

## Graphical abstract

**Figure:**
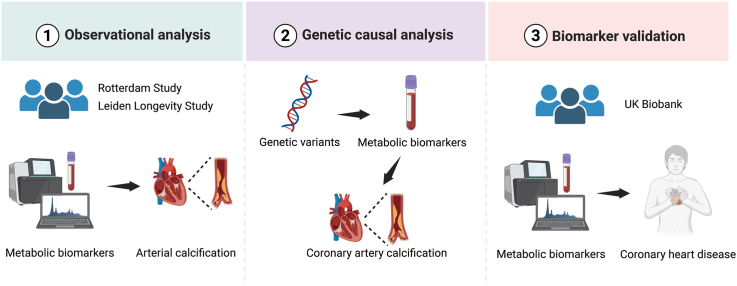

## 1 Introduction

Arterial calcification, a hallmark of arteriosclerosis, is a major key pathological driver for coronary heart disease (CHD). Although arterial calcification has a systemic nature, its burden varies significantly across different vascular beds, with moderate to high correlations being observed between them [1]. This implies that distinct risk factors and pathophysiological mechanisms may differentially influence calcification development across vessels. Furthermore, growing evidence points to significant sexual dimorphism in the pathophysiology of atherosclerosis, underscoring the need for deeper sex-stratified investigation [2].

Metabolic profiling of small-molecule biomarkers in biological systems is a powerful tool to investigate the biochemical pathways underlying complex traits. Alterations in biomarkers related to inflammation, lipid and carbohydrate metabolism, and oxidative stress have been implicated in the calcification of carotid arteries [3,4]. For example, circulating levels of ceramides, a biomarker involved in the pathways of inflammation, apoptosis, and cell death, have been shown as a predictor of CAC in patients with systemic lupus erythematosus [5]. The blood metabolic biomarkers, including 1,2-dipalmitoyl-sn-glycero-3-phosphocholine, glycohyocholate, and sphingomyelin, were also reported with genetic causal effects on vascular calcification [6]. Emerging evidence further suggests that glycolysis-related biomarkers exhibit distinct association patterns according to calcification sites and sex [4]. These metabolomic susceptibilities highlight the importance of in-depth investigation of metabolic mechanisms underlying the site- and sex-specific arterial calcification.

MR is an analytic method that investigates the genetic causal relationship underlying observational associations. Based on MR core assumptions, the valid genetic instruments in MR should meet the following requirements: (i) being associated with exposures; (ii) not being associated with any confounders; and (iii) influencing the outcome only through exposure [7]. The confounders' effects (both known or unknown) are an important source of bias in conventional observational studies; MR is robust to confounding effects as the genetic variants are randomly allocated at conception. Genome-wide association study (GWAS) have reported thousands of genetic variants associated with metabolic biomarkers and CAC [8,9], which can provide an opportunity to further investigate potential causal relationships between them.

This study examined the associations between targeted NMR-derived metabolic biomarkers and arterial calcification (CAC, AAC, and AVC) across population-based cohorts of RS and LLS, both overall and stratified by sex. Two-sample MR was then conducted to evaluate the potential causal nature of the observed associations. Finally, biomarkers exhibiting consistent observational and genetic evidence for CAC were validated by testing their association with CHD in UKBB.

## 2 Materials and methods

### 2.1 Study population

The Rotterdam Study (RS) is a prospective population-based study with participants from the Ommoord district in Rotterdam, the Netherlands [10]. It comprised over 17,000 participants aged 40 years and over, and included four sub-cohorts without any overlapping participants: RS-I (started in 1990, N = 7983), RS-II (started in 2000, N = 3011), RS-III (started in 2006, N = 3932), and RS-IV (started in 2017, N = 3005). RS invites participants for a regular examination every 3 to 4 years [11]. Blood sample was drawn after overnight fasting at each visit. This study utilized metabolic biomarkers measured from the fourth visit of RS-I (RS-I-4) and the third visit of RS-II (RS-II-3) [10]. Calcification from the multidetector computed tomography (MDCT) scan was available for the fourth visit of RS-I (RS-I-4) and the second visit of RS-II (RS-II-2). The mean interval between data collection for RS-II-2 (MDCT) and RS-II-3 (NMR metabolic biomarkers) was 5.86 years. RS-I and RS-II were considered as two separate datasets within the RS.

The Rotterdam Study has been approved by the Medical Ethics Committee of the Erasmus MC (registration number MEC 02.1015) and by the Dutch Ministry of Health, Welfare and Sport (Population Screening Act WBO, license number 1071272-159521-PG). The Rotterdam Study Personal Registration Data collection is filed with the Erasmus MC Data Protection Officer under registration number EMC1712001. The Rotterdam Study has been entered into the Dutch Trial Register (NTR; https://onderzoekmetmensen.nl) and into the WHO International Clinical Trials Registry Platform (ICTRP https://www.who.int/clinical-trials-registry-platform, search portal https://trialsearch.who.int/) under shared catalogue number NL6645/NTR6831. The Rotterdam Study project persistant identifier is https://ror.org/02ac58f22. All participants provided written informed consent to participate in the study and to have their information obtained from treating physicians.

The Leiden Longevity Study (LLS) enrolled individuals from 2002 to 2006, including long-lived family members (N = 1,671, mean age of 60 years) and their partners (N = 744, mean age of 60 years) as population controls [12]. Our study used participants from the inclusion period between 2009 and 2010, during which imaging data and Nightingale metabolic measurements were collected [13]. Blood samples were collected after an overnight fast. The LLS was approved by the medical ethics committee of the Leiden University Medical Center. All participants provided written informed consent.

The UK Biobank (UKBB) is a large population-based study that includes genetic data and a wide variety of phenotypes from over 500,000 participants aged 40 to 69 years at recruitment in the United Kingdom. Detailed protocols of UKBB can be found elsewhere [14]. Participants from the baseline assessment between 2006 and 2010 (referred to as instance 0 in UKBB) were included in this study. UK Biobank received ethical approval from the National Information Governance Board for Health and Social Care and the National Health Service North West Centre for Research Ethics Committee (Ref: 21/NW/0157), and all participants in this study have provided informed consent.

### 2.2 Metabolic biomarkers

Metabolic biomarkers in this study were measured from EDTA plasma samples using the Nightingale platform (Nightingale Health, Helsinki, Finland). This platform employs high-throughput proton NMR and enables simultaneous quantification of 249 metabolic biomarkers (168 absolute concentration measures and 81 biomarker ratios) [15]. As shown in Supplementary Table 1, most of the NMR biomarkers are related to lipoprotein metabolism, capturing lipid concentrations and composition across 14 lipoprotein subclasses, including triglycerides, phospholipids, total cholesterol, cholesterol esters, and free cholesterol, and total lipid concentration within each subclass [15]. In addition, the NMR panel also quantifies the absolute concentration and relative proportions of the abundant plasma fatty acids and small molecules. This platform provides highly reproducible and standardized measurements, making it suitable for large-scale population-based and epidemiological studies.

In RS, the R package of *Maplet* was used for quality control and pre-processing biomarker measurements [16]. Samples flagged as low protein content were excluded. Measurements with zero concentrations were considered missing. We excluded biomarkers with more than 40% missing values across samples and individual samples with more than 40% missing data for any measurements. Subsequently, biomarkers were log2-transformed to approximate normality. Missing values of the remaining measurements were then imputed from the k-nearest neighbors (kNN) algorithm. Sample outliers were detected and excluded from analysis using the local outlier factor method, followed by a second round of imputation for extreme outliers using the kNN.

In LLS, STATA/SE 16.1 (StataCorp. 2020. Stata Statistical Software: Release 16.1. College Station, TX: StataCorp LLC) was applied for dataset quality control, pre-processing, and analysis. Biomarkers with zero concentrations were treated as missing values, and samples with more than 10% missing values were removed. Subsequently, measurements were log2-transformed to approximate normality. We also used the kNN approach to impute missing values for the remaining biomarkers.

In UKBB, the recently released metabolic biomarkers from around 275,000 individuals were included in this study. Detailed protocols on biomarker collection, quantification, and quality control can be found elsewhere [17].

The metabolic biomarkers included in the analysis were measured as concentrations (mmol/L or g/L), percentages (%, for relative lipoprotein lipid concentrations and other ratios), and nanometers (nm, for biomarkers measuring lipid particle volume). We scaled the measurements into standard deviation (SD) units for subsequent analysis to facilitate comparison between cohorts.

### 2.3 Assessment of calcification

In RS, the 16-slice or 64-slice MDCT scanners (Somatom Sensation 16 or 64; Siemens, Forchheim, Germany) were used to obtain non-contrast CT images. The cardiac scan, along with an extra-cardiac scan that included the aortic root and aortic arch, was used to assess the calcification value from coronary artery, aortic arch, and aortic valve. Detailed information of the protocol, imaging settings, and the semi-automated method used for scoring calcifications is provided elsewhere [1,4]. Dedicated software (Syngo Calcium Scoring; Siemens, Forcheim, Germany) was used for the quantification of calcification. We further assessed the CAC based on the Agatston score, a standardized method for quantification of coronary calcium, for subsequent analyses [18]. Calcification volumes for AAC and AVC were expressed in cubic millimeters.

In LLS, non-contrast CT images between carina and cardiac apex region were obtained from 320-multidetector row CT scanner (Aquilion ONE, Toshiba, Otawara, Japan). More detailed descriptions of imaging settings can be found elsewhere [19]. Dedicated quantification software (VScore, Vital Images) was then used to semi-automatically measure the volumes of coronary artery calcification. A post-processing tool was used to automatically identify the areas of three or more contiguous voxels that exceeded the threshold value (130 HU). Subsequently, the calcified areas were manually encircled in the course of the coronary arteries. The Agatston score for each participant was calculated automatically based on the encircled areas. Imaging data analysis was performed blinded to group (offspring vs. partner) and clinical data. No measurement of AAC and AVC was available in LLS.

Considering the calcium volume measurements of zero, a constant of 1 was added prior to log-transformation as [Ln (calcification + 1.0)]. Both CAC, AAC, and AVC measures were subsequently scaled to SD units.

### 2.4 Covariates

Our study used relevant cardiovascular risk factors as covariates, including age, sex, diabetes mellitus, hypertension, hypercholesterolemia, current smoking status, body mass index (BMI), and history of cardiovascular disease (CVD). Seated blood pressure (BP) was measured twice in the right arm using a random-zero sphygmomanometer, with the mean value used for analysis. Hypertension was defined as systolic BP ≥ 140 mmHg, diastolic BP ≥ 90 mmHg, and/or antihypertensive medications. Glucose and lipids were measured using standard automated enzymatic procedures (Mannheim system for RS, Cobas system for LLS, and hexokinase analysis on a Beckman Coulter AU5800 in UKBB). We define diabetes in RS and LLS as plasma glucose ≥7 mmol/L or the use of diabetes medications [20]. For hypercholesterolemia, participants were identified as cases if their total cholesterol was larger than 6.2 mmol/L, or using lipid-lowering medication (RS and LLS) or cholesterol-lowering medication (UKBB) [20]. Information on current smoking and medication use was collected via home interviews for the RS or through questionnaires for LLS and UKBB. Standardized anthropometric assessments were conducted at the research facility by well-trained medical staff. BMI (kg/m^2^) was calculated from weight divided by height squared. CVD history was defined as previously diagnosed as CHD (myocardial infarction, coronary artery bypass graft, percutaneous transluminal coronary angioplasty), or stroke [4].

In UKBB, the covariate information was collected from baseline visit. BP was calculated from the mean value of two automated readings. As shown in Supplementary Table 2, both CHD, stroke, hypertension, and diabetes were defined by the combination of self-reported information, International Classification of Diseases version 9 (ICD-9) and 10 (ICD-10), and the Surveys Classification of Interventions and Procedures, version 4 (OPCS-4) [21].

### 2.5 Statistical analysis

#### 2.5.1 Association of metabolic biomarkers and calcification

To evaluate the associations between NMR metabolic biomarkers and calcification (CAC, AAC, and AVC), we conducted linear regression adjusted for age, sex, and lipid-lowering medication as the basic model (Model 1). Model 2 was additionally adjusted for diabetes mellitus, hypertension, hypercholesterolemia, current smoking status, BMI, and CVD history. Summary statistics from the RS-I, RS-II, and LLS were meta-analyzed using an inverse-variance weighted random-effects approach. Sex-stratified analyses were conducted using the same covariates as in Models 1 and 2, except for sex. Given the prior history of CVD may influence both metabolic biomarkers and calcification burden through medication use, inflammation, and metabolic remodeling, we additionally conducted sensitivity analyses by repeating Model 2 (both overall and sex-stratified) after excluding participants with prevalent CVD at baseline.

Given the high correlation among biomarkers, we applied the multiple testing correction method based on the effective number of independent tests (M_eff_), which accounts for the correlation structure among all biomarkers [22]. To maximize the included sample size, we used the biomarker correlation structure from RS-I to estimate the number of effectively independent tests, which yielded 38 independent biomarker features. Considering the three calcification outcomes investigated, we finally set the significance threshold as P-value < 4.39 × 10^−4^.

We also conducted a post-hoc statistical power analysis using the R package *pwr* (version 1.3.0, function pwr. f2. test). Detectable effect sizes were calculated assuming 80% statistical power, a multiple-testing adjusted significance threshold of 4.39 × 10^−4^, one predictor per outcome, and the available participants as the effective sample size.

All statistical analyses were performed using R software (version 4.3.2). The meta-analysis was performed using the R package: metafor (version 4.0.0).

#### 2.5.2 Mendelian randomization

We used two-sample MR to evaluate whether the identified associations [NMR metabolic biomarkers that were nominally (P-value < 0.05) associated with CAC] represent genetic causal effects. The biomarker-associated variants from GWAS in UKBB were used as genetic instrument variables (IVs) [8]. Detailed information on genetic instrument selection, harmonization, and F-statistic calculation was described in the Supplementary Materials.

We estimated the causal effect of each biomarker on CAC mainly using the inverse-variance-weighted (IVW) approach. It combines the Wald ratio effect for each IV (i.e., the coefficient of CAC divided by the coefficient of metabolic biomarkers) and could provide an unbiased estimate of causal effect if no IV has a horizontal pleiotropy effect [23,24]. Additionally, the weighted median (WM) and MR-Egger methods were also applied to estimate their causal effect, as they could provide a robust estimate in the presence of invalid IVs. Compared to IVW, MR-Egger regression is based on a similar approach but includes an intercept term, allowing for directional pleiotropy and providing a consistent estimate under the Instrument Strength Independent of Direct Effect (InSIDE) assumption [25].

As a sensitivity analysis, the MR-Egger test was used to assess evidence of horizontal pleiotropy for the remaining IVs. A nonzero intercept from the MR-Egger regression could indicate evidence of horizontal pleiotropy effects [25]. Besides, the IVs heterogeneity effect was also measured by Cochran's Q-statistic from IVW and MR-Egger regression.

Metabolic biomarkers were identified with genetic causal effects if they meet the following strict requirements: (1) significantly (Bonferroni correction considering the number of biomarkers tested) associated with CAC from the IVW, MR-Egger, or WM model, (2) with same direction as the observational association, (3) without evidence of horizontal pleiotropy from the MR Egger intercept method (P-value >0.05), (4) without evidence of heterogeneity effects from Cochran's Q-statistic (P-value >0.05).

Analysis from this section was performed by the R packages of TwoSampleMR [26] (version 0.5.6) and MRPRESSO [26] (version 1.0).

#### 2.5.3 Validation of biomarkers with CHD in UKBB

For biomarkers consistently and causally associated with CAC, we further validated their clinical role by investigating their association with prevalent CHD in UKBB. Logistic regression models were applied, adjusting for assessment center, age, sex, lipid-lowering medication, diabetes mellitus, hypertension, hypercholesterolemia, smoking status, and BMI. A Bonferroni correction, accounting for the number of biomarkers tested, was applied to determine the significant associations.

## 3 Results

### 3.1 Study characteristics

**Fig. 1:**
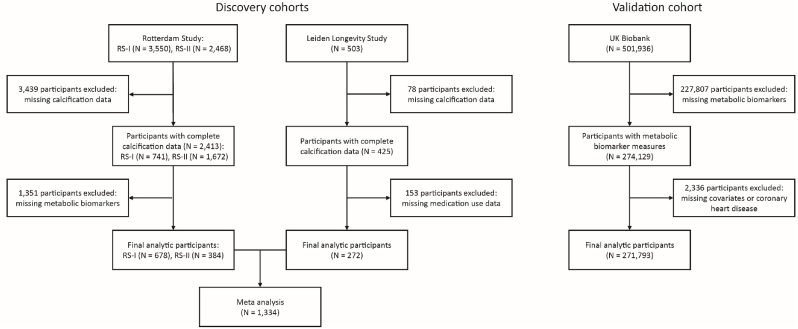
Flowchart of participant inclusion and exclusion.

The overall workflow indicating the inclusion and exclusion of participants in this study is illustrated in Fig. 1. A total of 1,334 (RS-I, RS-II, and LLS) and 271,793 (UKBB) participants were included in the discovery and validation analysis, respectively. Cohort-specific population characteristics were presented in Table 1. Participants from the RS-I were older (mean age of 73.21 years) and exhibited a higher prevalence of hypertension (81.12%), and CHD (9.59%) than those from RS-II (mean age of 64.48 years, 66.67%, and 3.39% respectively), LLS (mean age of 65.34 years, 64.79%, and 1.6%), and UKBB (mean age of 56.56 years, 30.34%, and 6.44%). The prevalence of individuals with CAC (CAC >0) was also higher in RS-I (86.73%), followed by RS-II (77.60%) and LLS (65.07%).

**Table 1:** Descriptive characteristics of the study cohorts.

	RS-I	RS-II	LLS	UKBB
Number of participants	678	384	272	271,793
Age in years, mean (SD)	73.21 (5.33)	64.48 (3.32)	65.34 (6.85)	56.56 (8.08)
Females (%)	329 (48.53)	206 (53.65)	134 (49.26)	146,747 (53.99)
Systolic blood pressure in mmHg, mean (SD)	151.78 (21.30)	142.39 (17.84)	141.56 (20.73)	137.87 (18.63)
Diastolic blood pressure in mmHg, mean (SD)	80.1 (11.30)	81.32 (10.36)	83.31 (10.67)	82.15 (10.15)
Hypertension (%)	550 (81.12)	256 (66.67)	138 (64.79)	82,475 (30.34)
Glucose in mmol/L, mean (SD)	5.85 (1.38)	5.59 (1.22)	5.46 (0.95)	5.12 (1.23)
Diabetes mellitus (%)	97 (14.31)	30 (7.81)	48 (17.65)	15,406 (5.67)
Total cholesterol in mmol/L, mean (SD)	5.62 (0.94)	5.78 (0.96)	5.45 (1.01)	5.69 (1.14)
HDL-cholesterol in mmol/L, mean (SD)	1.42 (0.37)	1.46 (0.40)	1.43 (0.35)	1.45 (0.38)
Hypercholesterolemia (%)	327 (48.23)	190 (49.48)	115 (42.44)	125,931 (46.33)
Current smoker (%)	81 (11.95)	58 (15.10)	40 (14.76)	28,691 (10.56)
BMI in kg/m2, mean (SD)	27.25 (3.78)	27.87 (3.81)	25.07 (3.17)	27.45 (4.78)
Prevalent cardiovascular disease (%)	82 (12.09)	23 (5.99)	8 (3.13)	17,515 (6.44)
Prevalent coronary heart disease (%)	65 (9.59)	13 (3.39)	4 (1.60)	17,515 (6.44)
Prevalent stroke (%)	20 (2.95)	11 (2.86)	5 (1.90)	5499 (2.02)
**Calcification measures**				
Coronary artery calcification Agatston score (median, IQR)	117.30 (11.75 - 506.50)	27.20 (0.55 - 164.40)	17.00 (0.00 - 164.50)	N.A.
Aortic arch calcification volume (median, IQR)	409.55 (119.28 - 1189.68)	92.25 (9.97 - 391.02)	N.A.	N.A.
Aortic valve calcification volume (median, IQR)	0.00 (0.00 - 54.40)	0.00 (0.00 - 0.00)	N.A.	N.A.
**Presence of calcification**				
Coronary artery calcification (%)	588 (86.73)	298 (77.60)	177 (65.07)	N.A.
Aortic arch calcification (%)	638 (94.10)	328 (85.42)	N.A.	N.A.
Aortic valve calcification (%)	300 (44.25)	79 (20.57)	N.A.	N.A.

BMI: body mass index; HDL: high-density lipoprotein; SD: standard deviation; IQR: interquartile range; N.A: not available.

Data collection periods: RS-I (2002-2004), RS-II (calcification from 2004 to 2005, and metabolic biomarkers from 2011 to 2012), LLS (2002-2006), and UKBB (2006-2010).

### 3.2 Metabolic biomarker profiles of CAC, AAC, and AVC

**Fig. 2:**
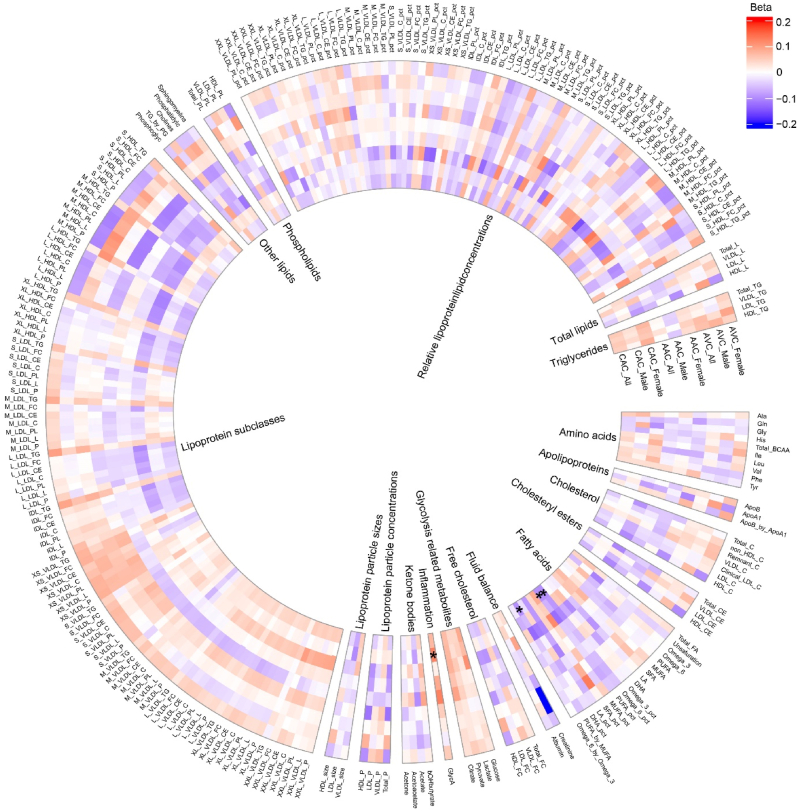
**Circos plot displaying associations (both in overall and sex-stratified) between metabolic biomarkers and vascular calcification at coronary artery (CAC), aortic arch (AAC), and aortic valve (AVC).** The color represents effect sizes (Beta) per standard deviation increase of metabolic biomarkers. Biomarkers and their groups are listed outside and inside the circle, respectively. Regression was adjusted by age, sex, lipid-lowering medication, hypertension, diabetes mellitus, hypercholesterolemia, smoking status, body mass index, and history of cardiovascular disease. Asterisk denotes significant associations after multiple testing correction (P-value < 4.39 × 10^−4^).

We next examined associations between 249 metabolic biomarkers and arterial calcification of CAC, AAC, and AVC in the overall study population (Fig. 2, Supplementary Tables 3 and 4). After adjustment for age, sex, and lipid-lowering medication (Supplementary Table 3), 14 biomarkers were significantly associated with either CAC or AAC, based on the multi-test correction of M_eff_ approach (P-value < 4.39 × 10^−4^). After further adjustment for additional covariates, including diabetes, hypertension, hypercholesterolemia, smoking status, BMI, and history of CVD (Model 2), 52, 5, and 4 biomarkers were nominally associated (P-value < 0.05) with CAC, AAC, and AVC, respectively (Supplementary Table 4). From multi-test correction, three ratio-based biomarkers from the fatty acids group remained significantly (P-value < 4.39 × 10^−4^) associated with CAC (Supplementary Table 4), including omega-6 fatty acids to total fatty acids (Omega_6_pct, Beta: −0.10, SE: 0.03, P-value: 2.94 × 10^−4^), polyunsaturated fatty acids to monounsaturated fatty acids (PUFA_by_MUFA, Beta: −0.10, SE: 0.03, P-value: 3.08 × 10^−4^), and polyunsaturated fatty acids to total fatty acids (PUFA_pct, Beta: −0.10, SE: 0.03, P-value: 3.33 × 10^−4^). No significant associations between biomarkers with AAC and AVC were observed.

### 3.3 Sex-stratified analyses

Results from the sex-stratified analyses are presented in Fig. 2, Supplementary Tables 5 and 6. In Model 1, we found glycoprotein acetyls (GlycA), a marker of inflammation, was significantly associated with AAC (Beta: 0.15, SE: 0.04, P-value: 2.71 × 10^−4^) and CAC (Beta: 0.12, SE: 0.03, P-value: 6.04 × 10^−4^) in males. Two biomarkers from the relative lipoprotein lipid concentrations group were significantly associated with CAC in females: cholesteryl esters to total lipids ratio in very small VLDL (Beta: −0.15, SE: 0.04, P-value: 1.96 × 10^−4^), and cholesterol to total lipids ratio in very small VLDL (Beta: −0.15, SE: 0.04, P-value: 2.12 × 10^−4^). After additional adjustment for cardiovascular risk factors in Model 2, only GlycA remained significantly associated with CAC in males (Beta: 0.13, SE: 0.04, P-value: 3.10 × 10^−4^).

### 3.4 Sensitivity analyses

Results of the sensitivity analysis by excluding participants with prevalent CVD are presented in Supplementary Table 7. PUFA_pct (Beta: −0.10, SE: 0.03, P-value: 3.50 × 10^−4^) and PUFA_by_MUFA (Beta: −0.10, SE: 0.03, P-value: 2.40 × 10^−4^) remain significantly and consistently associated with lower CAC. In addition, the cholesterol to total lipids ratio in small LDL (S_LDL_C_pct) was significantly associated with lower AAC burden (Beta: −0.12, SE: 0.03, P-value: 2.82 × 10^−4^).

Statistical power analysis (Supplementary Table 8) indicated that the study can be sufficiently powered to detect standardized effect sizes of approximately 0.12 for CAC and 0.14 for AAC/AVC in the overall sample. In sex-stratified analysis, detectable effect sizes were larger (0.17 to 0.20). The observed effect sizes in our analyses were smaller (particularly for AAC, AVC, and stratified analyses), suggesting that limited statistical power may have contributed to the largely null findings.

### 3.5 Mendelian randomization

We subsequently conducted two-sample MR to evaluate whether the associations between 52 biomarkers (P-value < 0.05) and CAC observed in the fully adjusted model reflect potential causal effects. The biomarker-associated variants used as IVs (after harmonization and outlier filter) in MR analyses are listed in Supplementary Table 9. Bonferroni correction accounting for 52 biomarkers (P-value < 9.62 × 10^−4^) was used for multi-test correction.

**Fig. 3:**
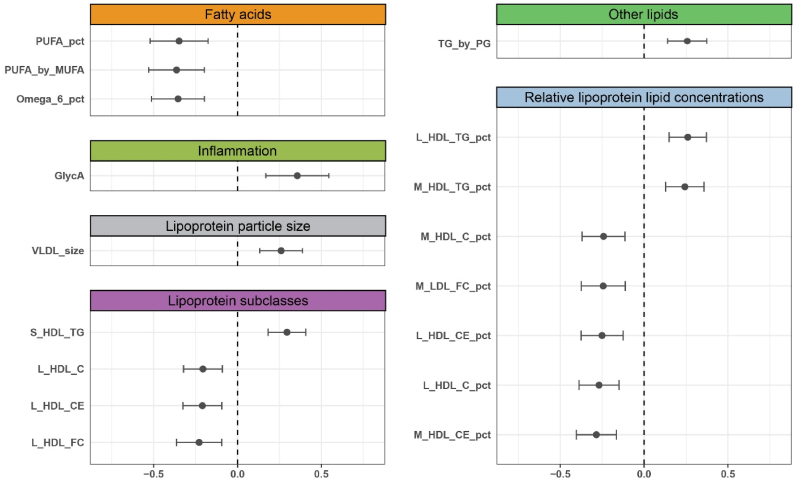
Forest plot indicating the causal associations between 17 metabolic biomarkers and coronary artery calcification from two-sample Mendelian randomization.

A total of 17 biomarkers met the strict requirements set and presented genetic evidence of causal effects, as shown in Fig. 3 and Supplementary Table 10. Those biomarkers were from 6 groups: Fatty acids (N = 3), Inflammation (N = 1), Lipoprotein particle size (N = 1), Lipoprotein subclasses (N = 4), Other lipids (N = 1), and Relative lipoprotein lipid concentrations (N = 7). The F-statistic larger than 10 indicates less evidence of weak instrument bias (Supplementary Table 10).

As shown in Supplementary Table 11, the sensitivity analysis suggested that MR causal estimation was less likely to be biased by pleiotropy and heterogeneity effects. Additionally, directionality tests supported a causal relationship from biomarkers to CAC.

### 3.6 Validation of metabolic biomarkers with CHD

We subsequently validated the 17 biomarkers that have consistent observational and genetic evidence with CAC by testing their association with clinical CHD in UKBB. From the Bonferroni correction accounting for 17 biomarkers tested (P-value < 2.94 × 10^−3^), 14 showed significant associations with CHD (Table 2).

**Table 2:** Validation of the 17 metabolic biomarkers and ratios in association with coronary heart disease (CHD) from the UK Biobank.

Group	Biomarkers and ratios	OR	lowCI	upCI	P-value
Fatty acids	PUFA_pct	1.00	0.99	1.02	0.58
	PUFA_by_MUFA	0.98	0.96	1.00	0.02
	Omega_6_pct	1.03	1.01	1.04	2.36 × 10^−3^
Inflammation	GlycA	1.00	0.98	1.02	0.96
Lipoprotein particle sizes	VLDL_size	0.95	0.94	0.97	1.03 × 10^−7^
Lipoprotein subclasses	S_HDL_TG	0.97	0.95	0.99	6.35 × 10^−4^
	L_HDL_C	0.81	0.79	0.83	1.22 × 10^−64^
	L_HDL_CE	0.81	0.79	0.83	1.02 × 10^−64^
	L_HDL_FC	0.82	0.80	0.84	1.57 × 10^−62^
Other lipids	TG_by_PG	1.07	1.05	1.08	1.61 × 10^−12^
Relative lipoprotein lipid concentrations	L_HDL_TG_pct	1.05	1.04	1.07	5.56 × 10^−13^
	M_HDL_TG_pct	1.09	1.07	1.11	3.74 × 10^−21^
	M_HDL_C_pct	0.88	0.87	0.90	2.54 × 10^−41^
	M_LDL_FC_pct	0.97	0.95	0.98	7.42 × 10^−5^
	L_HDL_CE_pct	0.95	0.93	0.96	3.34 × 10^−11^
	L_HDL_C_pct	0.95	0.93	0.96	1.14 × 10^−12^
	M_HDL_CE_pct	0.93	0.91	0.94	6.85 × 10^−17^

Groups: the NMR biomarker subgroups.

OR: Odd ratio, the odds change of CHD with one standard deviation increase of metabolic biomarker or ratios.

LowCI: lower 95% confidence interval of OR.

UpCI: upper 95% confidence interval of OR.

P-value: strength of evidence against the null hypothesis of no association between biomarker and CHD.

## 4 Discussion

This study investigated associations between NMR-derived metabolic biomarkers and arterial calcifications at multiple sites, both overall and stratified by sex. Notably, we observed significant associations between fatty acid ratio biomarkers and lower CAC burden, including Omega_6_pct, PUFA_by_MUFA, and PUFA_pct. Besides, the elevated level of GlycA was also positively associated with CAC in males only. Two-sample MR confirmed a consistent genetic causal effect between 17 biomarkers and CAC. Furthermore, the clinical relevance of these findings was underscored by the fact that 14 biomarkers were significantly associated with CHD in UKBB.

Unsaturated fatty acids, including MUFA and PUFA, are characterized by the presence of one or more double bonds in their hydrocarbon chains. Among PUFAs, omega-3 and omega-6 are biologically important classes and play key roles in atherosclerosis by modulating inflammatory processes. The protective effects of PUFA on vascular calcification and atherosclerosis are mediated by various mechanisms, including regulation of lipid metabolism and anti-inflammatory and antithrombotic pathways [27,28]. For example, omega-3 could lower triglycerides and act as an endogenous inhibitor of inflammation [29]. Experimental studies have demonstrated that omega-3 PUFAs exert multiple anti-atherogenic effects, including suppressing inflammatory cytokine production, reducing cell adhesion molecule expression, enhancing endothelial function, and improving plaque stability [30,31]. However, findings from randomized clinical trials have not consistently supported a beneficial effect of omega-3 on cardiovascular outcomes [30,32]. Similarly, our study found no evidence of association between omega-3 and CAC.

The role of dietary omega-6 on atherosclerosis pathogenesis also remains controversial. Although omega-6 fatty acids may exert pro-inflammatory effects, potentially increasing oxidative stress and promoting LDL oxidation *in vitro*, animal studies have also shown that omega-6 intake can reduce atherogenesis compared with control groups, even under pro-inflammatory conditions [33]. In our study, fatty acid ratio profiles enriched in PUFAs, particularly omega-6, were associated with lower CAC. These measures reflect the relative distribution of fatty-acid classes within the total circulating fatty-acid pool. Accordingly, higher values may result from increased PUFA or omega-6 levels, decreased MUFA or total fatty acid concentrations, or a combination of both. Moreover, circulating fatty acids are largely transported within lipoproteins; therefore, the ratio-based measures also reflect broader shifts in lipid metabolism and lipoprotein remodeling, including changes in particle composition, lipid exchange, and enzymatic processing. Consequently, the causal effect estimated from MR thus represents shared lipid metabolisms between numerator (PUFA or omega-6) and denominator (MUFA or total fatty acid) rather than isolated effects of individual fatty acids.

Furthermore, a higher omega-6 proportion was also associated with an increased risk of CHD. One potential explanation is that it could inhibit the calcification process or reduce the stable plaque calcification, while simultaneously promoting features of plaque vulnerability. Omega-6-enriched diets have been shown to reduce DNA synthesis in human coronary smooth muscle cells, a process crucial for atherosclerotic lesion progression [34]. Besides, through its roles in promoting oxidative stress, oxidized LDL formation, and chronic low-grade inflammation, omega-6 fatty acids may also act as drivers of CHD [35].

GlycA is an inflammation biomarker that summarizes the signals from glycan groups of certain acute-phase glycoproteins [36]. It has been reported to be associated with increased carotid artery calcification, predominantly in males [4]. Consistent with these findings, our study demonstrated that GlycA was associated with a larger CAC burden in males only. Inflammation plays a role in the initiation, progression, and complications of atherosclerosis. As a stable inflammatory biomarker, GlycA could capture distinct sources of inflammation, especially in younger individuals [37]. However, further research is warranted to elucidate the sex-specific mechanisms underlying the association between GlycA and vascular calcification.

We also found that lipoprotein and lipid ratio biomarkers, such as cholesterol and cholesteryl esters, were associated with both CAC and CHD. High-density lipoprotein (HDL) is a small, dense plasma lipoprotein that consists of several distinct particle subpopulations, including free cholesterol and cholesteryl esters. HDLs primarily transport cholesterol to the liver via both direct and indirect pathways, and have several potentially anti-atherogenic properties. It can remove cholesterol from cells, such as macrophages in the artery wall [38]. Besides, it could also inhibit low-density lipoprotein (LDL) oxidation [39], improve endothelial function, and have anti-thrombotic and anti-inflammatory properties [40]. Consistently, our study also found observational and genetic evidence for the protective effect of cholesterol and cholesteryl esters in large and medium HDL on CAC, which was further validated by their association with CHD. We also found that the triglycerides (TG) to total lipids ratio was associated with increased CAC and CHD risk. Plasma TG levels are synthesized through two primary ways: (1) the direct absorption of dietary lipids from the intestine, and (2) the hepatic *de novo* synthesis of triglycerides from circulating free fatty acids [41]. The excessive accumulation of plasma TG drives the pathological deposition of triglyceride-rich lipoproteins and their remnants in the arterial intima, a process that disrupts vascular homeostasis and contributes to the development of atherosclerosis [41]. Moreover, macrophages can incorporate cholesterol-enriched remnant particles, a by-product of TG metabolism, leading to foam cell formation in the arterial wall and subsequently promoting atherosclerosis [42].

A major strength of our study lies in the use of standardized metabolic profiling and the availability of calcification data across multiple vascular sites, enabling direct comparisons of associations between metabolic biomarkers and calcification across different arterial beds. The NMR approach provides highly reproducible and standardized measurements (primarily lipid, lipoprotein particle concentrations, and sizes) suitable for large-scale observational studies. Furthermore, the multi-cohort design enhances the generalizability and robustness of our findings. To support causal inference, we conducted MR analyses, which help mitigate bias from unmeasured confounding. However, several limitations also need to be discussed. First, the NMR panel predominantly captures lipid- and lipoprotein-related measures, with limited coverage of small molecules, thereby restricting the ability to identify novel metabolic pathways or subtle perturbations relevant to arterial calcification. In addition, causal interpretation of ratio-based biomarkers should be made with caution, as their genetic instruments likely reflect shared metabolic mechanisms linking the numerator and denominator rather than biomarker-specific biological effects. We also need to acknowledge that the MR signals (e.g., LDL-, HDL-, and GlycA biomarkers) represent established lipid and inflammatory mechanisms rather than indicating entirely novel pathways. The absence of detailed dietary intake data may have led to residual confounding of certain biomarker levels. It should also be acknowledged that the biomarkers and CT scan measures were not obtained at the same visit in RS-II, which could introduce variability. Although our overall sample size was substantial, statistical power was limited for detecting small effect sizes, particularly for AAC, AVC, and sex-stratification. Therefore, the largely null findings may reflect insufficient statistical power rather than the true absence of association. Future studies with larger sample sizes or pooled consortia are still needed.

In summary, this study identified NMR-derived metabolic biomarkers, particularly fatty acid ratios, that were significantly associated with lower CAC burden. These associations were further confirmed as a genetic causal relationship via MR analysis. Notably, the inflammation-related biomarker of GlycA was identified as a risk biomarker for CAC in males only. The subsequent validation of these biomarkers in association with CHD highlights their potential role in the disease pathogenesis.

## CRediT authorship contribution statement

**Haojie Lu:** Conceptualization, Formal analysis, Investigation, Writing – original draft, Writing – review & editing. **Maxime M. Bos:** Investigation, Writing – review & editing. **Nicolien A. van Vliet:** Writing – review & editing. **Mohsen Ghanbari:** Data curation, Writing – review & editing. **Marian Beekman:** Formal analysis, Writing – review & editing. **Luoshiyuan Zuo:** Writing – review & editing. **Pooja Mandaviya:** Writing – review & editing. **Dina Vojinovic:** Writing – review & editing. **Carolina Medina-Gomez:** Writing – review & editing. **Katerina Trajanoska:** Writing – review & editing. **P. Eline Slagboom:** Writing – review & editing. **Jeroen van der Grond:** Writing – review & editing. **M. Arfan Ikram:** Writing – review & editing. **Diana van Heemst:** Writing – review & editing. **Daniel Bos:** Data curation, Writing – review & editing. **Maryam Kavousi:** Conceptualization, Data curation, Funding acquisition, Resources, Supervision, Writing – review & editing. All authors approved the final manuscript.

## Financial disclosure

Metabolite measurements were funded by Biobanking and Biomolecular Resources Research Infrastructure (BBMRI)–NL (184.021.007) and the JNPD under the project PERADES (grant number 733051021, Defining Genetic, Polygenic and Environmental Risk for Alzheimer's Disease using multiple powerful cohorts, focused Epigenetics and Stem cell metabolomics). This work was supported by the project ‘B-specific’ funded by the European Union under Grant Agreement No. 101115159. This work was also supported by the Dutch Heart Foundation AtheroNeth consortium and the Leducq Foundation COMET Network. H.L is sponsored by the PhD fellowship (202004910412) from China Scholarship Council. K.T is supported by ERC-Advanced Grant LEGENDARE [project No. 101021500].

## Declaration of competing interest

The authors declare that they have no known competing financial interests or personal relationships that could have appeared to influence the work reported in this paper.

## Data Availability

In the Rotterdam Study and Leiden Longevity Study, the data set cannot be shared in public repository given the restrictions based on privacy regulations and informed consent of the participants. Data can be obtained upon reasonable request to the corresponding author. The phenotype datasets generated by the UK Biobank, analyzed during the current study, are available via the UK Biobank data access process (see http://www.ukbiobank.ac.uk/register-apply/).
