# Supplementary material for: Associations of NMR metabolic biomarkers and arterial calcification: An observational and Mendelian randomization study within the BBMRI metabolomics consortium
Source: Atheroscler Plus. 2026 Mar 10;64:24–32. doi: 10.1016/j.athplu.2026.03.001 (PMC13091362; doi:10.1016/j.athplu.2026.03.001)
Supplement: Multimedia component 1 [file mmc1.docx]

**Supplementary Materials**

**Mendelian randomization**

To remove the potential bias caused by the population structure, we focused on the genome-wide association (GWAS) of metabolomics and coronary artery calcification (CAC) from European ancestry in subsequent analysis. During the harmonization process, we flipped the effect and non-effect alleles for those IVs with conflicting direction (e.g., T/G for metabolic biomarkers and G/T for CAC) or strand issues (G/T for the metabolic biomarkers and C/A for the CAC) to make sure their effect size was referring to the same alleles^2^. Besides, we removed palindromic variants with intermediate allele frequency. To ensure no reversed causal effect, the MR Steiger filter was applied to remove variants with a reversed direction (explaining more variance in CAC than in metabolic biomarkers). We clumped IVs based on the r2 of 0.001 and windows of 10,000kb (with 1000 Genomes European ancestry as reference panel) to ensure all the included IVs are independent. To account for potential horizontal pleiotropy, we applied the MR-PRESSO (Mendelian Randomization Pleiotropy RESidual Sum and Outlier) approach to detect outlier IVs prior to the MR analyses^3^. IVs flagged as outliers by the MR-PRESSO outlier test were excluded for subsequent MR analysis.

The F-statistic was calculated based on the following formula to assess evidence of weak instrument bias in MR analysis^4^.

$$F =\frac{R^{2}\left( n-k-1 \right)}{k\left( 1-R^{2} \right)}$$

*n*, sample size included in the metabolites GWAS; *k* refers to the number of included IVs; *R^2^* is the proportion of metabolic biomarker variance explained by IVs. It can be calculated as *2×EAF×(1−EAF)×Beta^2^*, where *Beta* and *EAF* are the IVs’ effect size and effect allele frequency from metabolic biomarker GWAS. An F-statistic larger than 10 indicates less evidence of weak instrument bias^4^.

**References**

1. Richardson TG, Leyden GM, Wang Q, et al. Characterising metabolomic signatures of lipid-modifying therapies through drug target mendelian randomisation. *PLoS Biol.* 2022;20(2):e3001547.

2. Hemani G, Zheng J, Elsworth B, et al. The MR-Base platform supports systematic causal inference across the human phenome. *Elife.* 2018;7.

3. Wu F, Huang Y, Hu J, Shao Z. Mendelian randomization study of inflammatory bowel disease and bone mineral density. *BMC Med.* 2020;18(1):312.

4. Burgess S, Thompson SG, Collaboration CCG. Avoiding bias from weak instruments in Mendelian randomization studies. *Int J Epidemiol.* 2011;40(3):755-764.
