# Supplementary material for: Associations of NMR metabolic biomarkers and arterial calcification: An observational and Mendelian randomization study within the BBMRI metabolomics consortium
Source: Atheroscler Plus. 2026 Mar 10;64:24–32. doi: 10.1016/j.athplu.2026.03.001 (PMC13091362; doi:10.1016/j.athplu.2026.03.001)
Supplement: Multimedia component 2 [file mmc2.docx]

**Supplementary Tables**

**Supplementary Table 1:** Nightingale metabolic biomarkers annotation.

**Supplementary Table 2:** Disease definition code from UK Biobank.

**Supplementary Table 3:** Nominally significant association (P-value < 0.05) between NMR metabolic biomarkers and calcification in the overall population from Model 1.

**Supplementary Table 4:** Nominally significant association (P-value < 0.05) between NMR metabolic biomarkers and calcification in the overall population from model2.

**Supplementary Table 5:** Nominally significant association (P-value < 0.05) between NMR metabolic biomarkers and calcification in sex-stratified analysis (male only).

**Supplementary Table 6:** Nominally significant association (P-value < 0.05) between NMR metabolic biomarkers and calcification in sex-stratified analysis (female only).

**Supplementary Table 7:** Metabolic biomarkers significantly associated with calcification from sensitivity analysis by excluding participants with history of cardiovascular diseases.

**Supplementary Table 8:** Post-hoc sensitivity analysis of detectable standardized effect sizes for calcification at coronary artery (CAC), aortic arch (AAC), and aortic valve (AVC).

**Supplementary Table 9:** The variants included as genetic instruments in two-sample Mendelian randomization.

**Supplementary Table 10.** Two-sample Mendelian randomization (MR) indicates the causal associations between NMR 17 metabolic biomarkers and coronary artery calcification.

**Supplementary Table 11:** The sensitivity analysis of two-sample Mendelian randomization.

**Supplementary Table 1:** **Nightingale metabolic biomarkers annotation**

| **Metabolic biomarkers** | **Biomarker name** | **Unit** | **Metabolic groups** |
| --- | --- | --- | --- |
| Total_C | Total cholesterol | mmol/l | Cholesterol |
| non_HDL_C | Total cholesterol minus HDL-C | mmol/l | Cholesterol |
| Remnant_C | Remnant cholesterol (non-HDL, non-LDL -cholesterol) | mmol/l | Cholesterol |
| VLDL_C | VLDL cholesterol | mmol/l | Cholesterol |
| Clinical_LDL_C | Clinical LDL cholesterol | mmol/l | Cholesterol |
| LDL_C | LDL cholesterol | mmol/l | Cholesterol |
| HDL_C | HDL cholesterol | mmol/l | Cholesterol |
| Total_TG | Total triglycerides | mmol/l | Triglycerides |
| VLDL_TG | Triglycerides in VLDL | mmol/l | Triglycerides |
| LDL_TG | Triglycerides in LDL | mmol/l | Triglycerides |
| HDL_TG | Triglycerides in HDL | mmol/l | Triglycerides |
| Total_PL | Total phospholipids in lipoprotein particles | mmol/l | Phospholipids |
| VLDL_PL | Phospholipids in VLDL | mmol/l | Phospholipids |
| LDL_PL | Phospholipids in LDL | mmol/l | Phospholipids |
| HDL_PL | Phospholipids in HDL | mmol/l | Phospholipids |
| Total_CE | Total esterified cholesterol | mmol/l | Cholesteryl esters |
| VLDL_CE | Cholesteryl esters in VLDL | mmol/l | Cholesteryl esters |
| LDL_CE | Cholesteryl esters in LDL | mmol/l | Cholesteryl esters |
| HDL_CE | Cholesteryl esters in HDL | mmol/l | Cholesteryl esters |
| Total_FC | Total free cholesterol | mmol/l | Free cholesterol |
| VLDL_FC | Free cholesterol in VLDL | mmol/l | Free cholesterol |
| LDL_FC | Free cholesterol in LDL | mmol/l | Free cholesterol |
| HDL_FC | Free cholesterol in HDL | mmol/l | Free cholesterol |
| Total_L | Total lipids in lipoprotein particles | mmol/l | Total lipids |
| VLDL_L | Total lipids in VLDL | mmol/l | Total lipids |
| LDL_L | Total lipids in LDL | mmol/l | Total lipids |
| HDL_L | Total lipids in HDL | mmol/l | Total lipids |
| Total_P | Total concentration of lipoprotein particles | mmol/l | Lipoprotein particle concentrations |
| VLDL_P | Concentration of VLDL particles | mmol/l | Lipoprotein particle concentrations |
| LDL_P | Concentration of LDL particles | mmol/l | Lipoprotein particle concentrations |
| HDL_P | Concentration of HDL particles | mmol/l | Lipoprotein particle concentrations |
| VLDL_size | Average diameter for VLDL particles | nm | Lipoprotein particle sizes |
| LDL_size | Average diameter for LDL particles | nm | Lipoprotein particle sizes |
| HDL_size | Average diameter for HDL particles | nm | Lipoprotein particle sizes |
| Phosphoglyc | Phosphoglycerides | mmol/l | Other lipids |
| TG_by_PG | Ratio of triglycerides to phosphoglycerides | ratio | Other lipids |
| Cholines | Total cholines | mmol/l | Other lipids |
| Phosphatidylc | Phosphatidylcholines | mmol/l | Other lipids |
| Sphingomyelins | Sphingomyelins | mmol/l | Other lipids |
| ApoB | Apolipoprotein B | g/l | Apolipoproteins |
| ApoA1 | Apolipoprotein A1 | g/l | Apolipoproteins |
| ApoB_by_ApoA1 | Ratio of apolipoprotein B to apolipoprotein A1 | ratio | Apolipoproteins |
| Total_FA | Total fatty acids | mmol/l | Fatty acids |
| Unsaturation | Degree of unsaturation | degree | Fatty acids |
| Omega_3 | Omega-3 fatty acids | mmol/l | Fatty acids |
| Omega_6 | Omega-6 fatty acids | mmol/l | Fatty acids |
| PUFA | Polyunsaturated fatty acids | mmol/l | Fatty acids |
| MUFA | Monounsaturated fatty acids | mmol/l | Fatty acids |
| SFA | Saturated fatty acids | mmol/l | Fatty acids |
| LA | Linoleic acid | mmol/l | Fatty acids |
| DHA | Docosahexaenoic acid | mmol/l | Fatty acids |
| Omega_3_pct | Ratio of omega-3 fatty acids to total fatty acids | % | Fatty acids |
| Omega_6_pct | Ratio of omega-6 fatty acids to total fatty acids | % | Fatty acids |
| PUFA_pct | Ratio of polyunsaturated fatty acids to total fatty acids | % | Fatty acids |
| MUFA_pct | Ratio of monounsaturated fatty acids to total fatty acids | % | Fatty acids |
| SFA_pct | Ratio of saturated fatty acids to total fatty acids | % | Fatty acids |
| LA_pct | Ratio of linoleic acid to total fatty acids | % | Fatty acids |
| DHA_pct | Ratio of docosahexaenoic acid to total fatty acids | % | Fatty acids |
| PUFA_by_MUFA | Ratio of polyunsaturated fatty acids to monounsaturated fatty acids | ratio | Fatty acids |
| Omega_6_by_Omega_3 | Ratio of omega-6 fatty acids to omega-3 fatty acids | ratio | Fatty acids |
| Ala | Alanine | mmol/l | Amino acids |
| Gln | Glutamine | mmol/l | Amino acids |
| Gly | Glycine | mmol/l | Amino acids |
| His | Histidine | mmol/l | Amino acids |
| Total_BCAA | Total concentration of branched-chain amino acids (leucine + isoleucine + valine) | mmol/l | Amino acids |
| Ile | Isoleucine | mmol/l | Amino acids |
| Leu | Leucine | mmol/l | Amino acids |
| Val | Valine | mmol/l | Amino acids |
| Phe | Phenylalanine | mmol/l | Amino acids |
| Tyr | Tyrosine | mmol/l | Amino acids |
| Glucose | Glucose | mmol/l | Glycolysis related metabolites |
| Lactate | Lactate | mmol/l | Glycolysis related metabolites |
| Pyruvate | Pyruvate | mmol/l | Glycolysis related metabolites |
| Citrate | Citrate | mmol/l | Glycolysis related metabolites |
| bOHbutyrate | 3-Hydroxybutyrate | mmol/l | Ketone bodies |
| Acetate | Acetate | mmol/l | Ketone bodies |
| Acetoacetate | Acetoacetate | mmol/l | Ketone bodies |
| Acetone | Acetone | mmol/l | Ketone bodies |
| Creatinine | Creatinine | μmol/l | Fluid balance |
| Albumin | Albumin | g/l | Fluid balance |
| GlycA | Glycoprotein acetyls | mmol/l | Inflammation |
| XXL_VLDL_P | Concentration of chylomicrons and extremely large VLDL particles | mmol/l | Lipoprotein subclasses |
| XXL_VLDL_L | Total lipids in chylomicrons and extremely large VLDL | mmol/l | Lipoprotein subclasses |
| XXL_VLDL_PL | Phospholipids in chylomicrons and extremely large VLDL | mmol/l | Lipoprotein subclasses |
| XXL_VLDL_C | Cholesterol in chylomicrons and extremely large VLDL | mmol/l | Lipoprotein subclasses |
| XXL_VLDL_CE | Cholesteryl esters in chylomicrons and extremely large VLDL | mmol/l | Lipoprotein subclasses |
| XXL_VLDL_FC | Free cholesterol in chylomicrons and extremely large VLDL | mmol/l | Lipoprotein subclasses |
| XXL_VLDL_TG | Triglycerides in chylomicrons and extremely large VLDL | mmol/l | Lipoprotein subclasses |
| XL_VLDL_P | Concentration of very large VLDL particles | mmol/l | Lipoprotein subclasses |
| XL_VLDL_L | Total lipids in very large VLDL | mmol/l | Lipoprotein subclasses |
| XL_VLDL_PL | Phospholipids in very large VLDL | mmol/l | Lipoprotein subclasses |
| XL_VLDL_C | Cholesterol in very large VLDL | mmol/l | Lipoprotein subclasses |
| XL_VLDL_CE | Cholesteryl esters in very large VLDL | mmol/l | Lipoprotein subclasses |
| XL_VLDL_FC | Free cholesterol in very large VLDL | mmol/l | Lipoprotein subclasses |
| XL_VLDL_TG | Triglycerides in very large VLDL | mmol/l | Lipoprotein subclasses |
| L_VLDL_P | Concentration of large VLDL particles | mmol/l | Lipoprotein subclasses |
| L_VLDL_L | Total lipids in large VLDL | mmol/l | Lipoprotein subclasses |
| L_VLDL_PL | Phospholipids in large VLDL | mmol/l | Lipoprotein subclasses |
| L_VLDL_C | Cholesterol in large VLDL | mmol/l | Lipoprotein subclasses |
| L_VLDL_CE | Cholesteryl esters in large VLDL | mmol/l | Lipoprotein subclasses |
| L_VLDL_FC | Free cholesterol in large VLDL | mmol/l | Lipoprotein subclasses |
| L_VLDL_TG | Triglycerides in large VLDL | mmol/l | Lipoprotein subclasses |
| M_VLDL_P | Concentration of medium VLDL particles | mmol/l | Lipoprotein subclasses |
| M_VLDL_L | Total lipids in medium VLDL | mmol/l | Lipoprotein subclasses |
| M_VLDL_PL | Phospholipids in medium VLDL | mmol/l | Lipoprotein subclasses |
| M_VLDL_C | Cholesterol in medium VLDL | mmol/l | Lipoprotein subclasses |
| M_VLDL_CE | Cholesteryl esters in medium VLDL | mmol/l | Lipoprotein subclasses |
| M_VLDL_FC | Free cholesterol in medium VLDL | mmol/l | Lipoprotein subclasses |
| M_VLDL_TG | Triglycerides in medium VLDL | mmol/l | Lipoprotein subclasses |
| S_VLDL_P | Concentration of small VLDL particles | mmol/l | Lipoprotein subclasses |
| S_VLDL_L | Total lipids in small VLDL | mmol/l | Lipoprotein subclasses |
| S_VLDL_PL | Phospholipids in small VLDL | mmol/l | Lipoprotein subclasses |
| S_VLDL_C | Cholesterol in small VLDL | mmol/l | Lipoprotein subclasses |
| S_VLDL_CE | Cholesteryl esters in small VLDL | mmol/l | Lipoprotein subclasses |
| S_VLDL_FC | Free cholesterol in small VLDL | mmol/l | Lipoprotein subclasses |
| S_VLDL_TG | Triglycerides in small VLDL | mmol/l | Lipoprotein subclasses |
| XS_VLDL_P | Concentration of very small VLDL particles | mmol/l | Lipoprotein subclasses |
| XS_VLDL_L | Total lipids in very small VLDL | mmol/l | Lipoprotein subclasses |
| XS_VLDL_PL | Phospholipids in very small VLDL | mmol/l | Lipoprotein subclasses |
| XS_VLDL_C | Cholesterol in very small VLDL | mmol/l | Lipoprotein subclasses |
| XS_VLDL_CE | Cholesteryl esters in very small VLDL | mmol/l | Lipoprotein subclasses |
| XS_VLDL_FC | Free cholesterol in very small VLDL | mmol/l | Lipoprotein subclasses |
| XS_VLDL_TG | Triglycerides in very small VLDL | mmol/l | Lipoprotein subclasses |
| IDL_P | Concentration of IDL particles | mmol/l | Lipoprotein subclasses |
| IDL_L | Total lipids in IDL | mmol/l | Lipoprotein subclasses |
| IDL_PL | Phospholipids in IDL | mmol/l | Lipoprotein subclasses |
| IDL_C | Cholesterol in IDL | mmol/l | Lipoprotein subclasses |
| IDL_CE | Cholesteryl esters in IDL | mmol/l | Lipoprotein subclasses |
| IDL_FC | Free cholesterol in IDL | mmol/l | Lipoprotein subclasses |
| IDL_TG | Triglycerides in IDL | mmol/l | Lipoprotein subclasses |
| L_LDL_P | Concentration of large LDL particles | mmol/l | Lipoprotein subclasses |
| L_LDL_L | Total lipids in large LDL | mmol/l | Lipoprotein subclasses |
| L_LDL_PL | Phospholipids in large LDL | mmol/l | Lipoprotein subclasses |
| L_LDL_C | Cholesterol in large LDL | mmol/l | Lipoprotein subclasses |
| L_LDL_CE | Cholesteryl esters in large LDL | mmol/l | Lipoprotein subclasses |
| L_LDL_FC | Free cholesterol in large LDL | mmol/l | Lipoprotein subclasses |
| L_LDL_TG | Triglycerides in large LDL | mmol/l | Lipoprotein subclasses |
| M_LDL_P | Concentration of medium LDL particles | mmol/l | Lipoprotein subclasses |
| M_LDL_L | Total lipids in medium LDL | mmol/l | Lipoprotein subclasses |
| M_LDL_PL | Phospholipids in medium LDL | mmol/l | Lipoprotein subclasses |
| M_LDL_C | Cholesterol in medium LDL | mmol/l | Lipoprotein subclasses |
| M_LDL_CE | Cholesteryl esters in medium LDL | mmol/l | Lipoprotein subclasses |
| M_LDL_FC | Free cholesterol in medium LDL | mmol/l | Lipoprotein subclasses |
| M_LDL_TG | Triglycerides in medium LDL | mmol/l | Lipoprotein subclasses |
| S_LDL_P | Concentration of small LDL particles | mmol/l | Lipoprotein subclasses |
| S_LDL_L | Total lipids in small LDL | mmol/l | Lipoprotein subclasses |
| S_LDL_PL | Phospholipids in small LDL | mmol/l | Lipoprotein subclasses |
| S_LDL_C | Cholesterol in small LDL | mmol/l | Lipoprotein subclasses |
| S_LDL_CE | Cholesteryl esters in small LDL | mmol/l | Lipoprotein subclasses |
| S_LDL_FC | Free cholesterol in small LDL | mmol/l | Lipoprotein subclasses |
| S_LDL_TG | Triglycerides in small LDL | mmol/l | Lipoprotein subclasses |
| XL_HDL_P | Concentration of very large HDL particles | mmol/l | Lipoprotein subclasses |
| XL_HDL_L | Total lipids in very large HDL | mmol/l | Lipoprotein subclasses |
| XL_HDL_PL | Phospholipids in very large HDL | mmol/l | Lipoprotein subclasses |
| XL_HDL_C | Cholesterol in very large HDL | mmol/l | Lipoprotein subclasses |
| XL_HDL_CE | Cholesteryl esters in very large HDL | mmol/l | Lipoprotein subclasses |
| XL_HDL_FC | Free cholesterol in very large HDL | mmol/l | Lipoprotein subclasses |
| XL_HDL_TG | Triglycerides in very large HDL | mmol/l | Lipoprotein subclasses |
| L_HDL_P | Concentration of large HDL particles | mmol/l | Lipoprotein subclasses |
| L_HDL_L | Total lipids in large HDL | mmol/l | Lipoprotein subclasses |
| L_HDL_PL | Phospholipids in large HDL | mmol/l | Lipoprotein subclasses |
| L_HDL_C | Cholesterol in large HDL | mmol/l | Lipoprotein subclasses |
| L_HDL_CE | Cholesteryl esters in large HDL | mmol/l | Lipoprotein subclasses |
| L_HDL_FC | Free cholesterol in large HDL | mmol/l | Lipoprotein subclasses |
| L_HDL_TG | Triglycerides in large HDL | mmol/l | Lipoprotein subclasses |
| M_HDL_P | Concentration of medium HDL particles | mmol/l | Lipoprotein subclasses |
| M_HDL_L | Total lipids in medium HDL | mmol/l | Lipoprotein subclasses |
| M_HDL_PL | Phospholipids in medium HDL | mmol/l | Lipoprotein subclasses |
| M_HDL_C | Cholesterol in medium HDL | mmol/l | Lipoprotein subclasses |
| M_HDL_CE | Cholesteryl esters in medium HDL | mmol/l | Lipoprotein subclasses |
| M_HDL_FC | Free cholesterol in medium HDL | mmol/l | Lipoprotein subclasses |
| M_HDL_TG | Triglycerides in medium HDL | mmol/l | Lipoprotein subclasses |
| S_HDL_P | Concentration of small HDL particles | mmol/l | Lipoprotein subclasses |
| S_HDL_L | Total lipids in small HDL | mmol/l | Lipoprotein subclasses |
| S_HDL_PL | Phospholipids in small HDL | mmol/l | Lipoprotein subclasses |
| S_HDL_C | Cholesterol in small HDL | mmol/l | Lipoprotein subclasses |
| S_HDL_CE | Cholesteryl esters in small HDL | mmol/l | Lipoprotein subclasses |
| S_HDL_FC | Free cholesterol in small HDL | mmol/l | Lipoprotein subclasses |
| S_HDL_TG | Triglycerides in small HDL | mmol/l | Lipoprotein subclasses |
| XXL_VLDL_PL_pct | Phospholipids to total lipids ratio in chylomicrons and extremely large VLDL | % | Relative lipoprotein lipid concentrations |
| XXL_VLDL_C_pct | Cholesterol to total lipids ratio in chylomicrons and extremely large VLDL | % | Relative lipoprotein lipid concentrations |
| XXL_VLDL_CE_pct | Cholesteryl esters to total lipids ratio in chylomicrons and extremely large VLDL | % | Relative lipoprotein lipid concentrations |
| XXL_VLDL_FC_pct | Free cholesterol to total lipids ratio in chylomicrons and extremely large VLDL | % | Relative lipoprotein lipid concentrations |
| XXL_VLDL_TG_pct | Triglycerides to total lipids ratio in chylomicrons and extremely large VLDL | % | Relative lipoprotein lipid concentrations |
| XL_VLDL_PL_pct | Phospholipids to total lipids ratio in very large VLDL | % | Relative lipoprotein lipid concentrations |
| XL_VLDL_C_pct | Cholesterol to total lipids ratio in very large VLDL | % | Relative lipoprotein lipid concentrations |
| XL_VLDL_CE_pct | Cholesteryl esters to total lipids ratio in very large VLDL | % | Relative lipoprotein lipid concentrations |
| XL_VLDL_FC_pct | Free cholesterol to total lipids ratio in very large VLDL | % | Relative lipoprotein lipid concentrations |
| XL_VLDL_TG_pct | Triglycerides to total lipids ratio in very large VLDL | % | Relative lipoprotein lipid concentrations |
| L_VLDL_PL_pct | Phospholipids to total lipids ratio in large VLDL | % | Relative lipoprotein lipid concentrations |
| L_VLDL_C_pct | Cholesterol to total lipids ratio in large VLDL | % | Relative lipoprotein lipid concentrations |
| L_VLDL_CE_pct | Cholesteryl esters to total lipids ratio in large VLDL | % | Relative lipoprotein lipid concentrations |
| L_VLDL_FC_pct | Free cholesterol to total lipids ratio in large VLDL | % | Relative lipoprotein lipid concentrations |
| L_VLDL_TG_pct | Triglycerides to total lipids ratio in large VLDL | % | Relative lipoprotein lipid concentrations |
| M_VLDL_PL_pct | Phospholipids to total lipids ratio in medium VLDL | % | Relative lipoprotein lipid concentrations |
| M_VLDL_C_pct | Cholesterol to total lipids ratio in medium VLDL | % | Relative lipoprotein lipid concentrations |
| M_VLDL_CE_pct | Cholesteryl esters to total lipids ratio in medium VLDL | % | Relative lipoprotein lipid concentrations |
| M_VLDL_FC_pct | Free cholesterol to total lipids ratio in medium VLDL | % | Relative lipoprotein lipid concentrations |
| M_VLDL_TG_pct | Triglycerides to total lipids ratio in medium VLDL | % | Relative lipoprotein lipid concentrations |
| S_VLDL_PL_pct | Phospholipids to total lipids ratio in small VLDL | % | Relative lipoprotein lipid concentrations |
| S_VLDL_C_pct | Cholesterol to total lipids ratio in small VLDL | % | Relative lipoprotein lipid concentrations |
| S_VLDL_CE_pct | Cholesteryl esters to total lipids ratio in small VLDL | % | Relative lipoprotein lipid concentrations |
| S_VLDL_FC_pct | Free cholesterol to total lipids ratio in small VLDL | % | Relative lipoprotein lipid concentrations |
| S_VLDL_TG_pct | Triglycerides to total lipids ratio in small VLDL | % | Relative lipoprotein lipid concentrations |
| XS_VLDL_PL_pct | Phospholipids to total lipids ratio in very small VLDL | % | Relative lipoprotein lipid concentrations |
| XS_VLDL_C_pct | Cholesterol to total lipids ratio in very small VLDL | % | Relative lipoprotein lipid concentrations |
| XS_VLDL_CE_pct | Cholesteryl esters to total lipids ratio in very small VLDL | % | Relative lipoprotein lipid concentrations |
| XS_VLDL_FC_pct | Free cholesterol to total lipids ratio in very small VLDL | % | Relative lipoprotein lipid concentrations |
| XS_VLDL_TG_pct | Triglycerides to total lipids ratio in very small VLDL | % | Relative lipoprotein lipid concentrations |
| IDL_PL_pct | Phospholipids to total lipids ratio in IDL | % | Relative lipoprotein lipid concentrations |
| IDL_C_pct | Cholesterol to total lipids ratio in IDL | % | Relative lipoprotein lipid concentrations |
| IDL_CE_pct | Cholesteryl esters to total lipids ratio in IDL | % | Relative lipoprotein lipid concentrations |
| IDL_FC_pct | Free cholesterol to total lipids ratio in IDL | % | Relative lipoprotein lipid concentrations |
| IDL_TG_pct | Triglycerides to total lipids ratio in IDL | % | Relative lipoprotein lipid concentrations |
| L_LDL_PL_pct | Phospholipids to total lipids ratio in large LDL | % | Relative lipoprotein lipid concentrations |
| L_LDL_C_pct | Cholesterol to total lipids ratio in large LDL | % | Relative lipoprotein lipid concentrations |
| L_LDL_CE_pct | Cholesteryl esters to total lipids ratio in large LDL | % | Relative lipoprotein lipid concentrations |
| L_LDL_FC_pct | Free cholesterol to total lipids ratio in large LDL | % | Relative lipoprotein lipid concentrations |
| L_LDL_TG_pct | Triglycerides to total lipids ratio in large LDL | % | Relative lipoprotein lipid concentrations |
| M_LDL_PL_pct | Phospholipids to total lipids ratio in medium LDL | % | Relative lipoprotein lipid concentrations |
| M_LDL_C_pct | Cholesterol to total lipids ratio in medium LDL | % | Relative lipoprotein lipid concentrations |
| M_LDL_CE_pct | Cholesteryl esters to total lipids ratio in medium LDL | % | Relative lipoprotein lipid concentrations |
| M_LDL_FC_pct | Free cholesterol to total lipids ratio in medium LDL | % | Relative lipoprotein lipid concentrations |
| M_LDL_TG_pct | Triglycerides to total lipids ratio in medium LDL | % | Relative lipoprotein lipid concentrations |
| S_LDL_PL_pct | Phospholipids to total lipids ratio in small LDL | % | Relative lipoprotein lipid concentrations |
| S_LDL_C_pct | Cholesterol to total lipids ratio in small LDL | % | Relative lipoprotein lipid concentrations |
| S_LDL_CE_pct | Cholesteryl esters to total lipids ratio in small LDL | % | Relative lipoprotein lipid concentrations |
| S_LDL_FC_pct | Free cholesterol to total lipids ratio in small LDL | % | Relative lipoprotein lipid concentrations |
| S_LDL_TG_pct | Triglycerides to total lipids ratio in small LDL | % | Relative lipoprotein lipid concentrations |
| XL_HDL_PL_pct | Phospholipids to total lipids ratio in very large HDL | % | Relative lipoprotein lipid concentrations |
| XL_HDL_C_pct | Cholesterol to total lipids ratio in very large HDL | % | Relative lipoprotein lipid concentrations |
| XL_HDL_CE_pct | Cholesteryl esters to total lipids ratio in very large HDL | % | Relative lipoprotein lipid concentrations |
| XL_HDL_FC_pct | Free cholesterol to total lipids ratio in very large HDL | % | Relative lipoprotein lipid concentrations |
| XL_HDL_TG_pct | Triglycerides to total lipids ratio in very large HDL | % | Relative lipoprotein lipid concentrations |
| L_HDL_PL_pct | Phospholipids to total lipids ratio in large HDL | % | Relative lipoprotein lipid concentrations |
| L_HDL_C_pct | Cholesterol to total lipids ratio in large HDL | % | Relative lipoprotein lipid concentrations |
| L_HDL_CE_pct | Cholesteryl esters to total lipids ratio in large HDL | % | Relative lipoprotein lipid concentrations |
| L_HDL_FC_pct | Free cholesterol to total lipids ratio in large HDL | % | Relative lipoprotein lipid concentrations |
| L_HDL_TG_pct | Triglycerides to total lipids ratio in large HDL | % | Relative lipoprotein lipid concentrations |
| M_HDL_PL_pct | Phospholipids to total lipids ratio in medium HDL | % | Relative lipoprotein lipid concentrations |
| M_HDL_C_pct | Cholesterol to total lipids ratio in medium HDL | % | Relative lipoprotein lipid concentrations |
| M_HDL_CE_pct | Cholesteryl esters to total lipids ratio in medium HDL | % | Relative lipoprotein lipid concentrations |
| M_HDL_FC_pct | Free cholesterol to total lipids ratio in medium HDL | % | Relative lipoprotein lipid concentrations |
| M_HDL_TG_pct | Triglycerides to total lipids ratio in medium HDL | % | Relative lipoprotein lipid concentrations |
| S_HDL_PL_pct | Phospholipids to total lipids ratio in small HDL | % | Relative lipoprotein lipid concentrations |
| S_HDL_C_pct | Cholesterol to total lipids ratio in small HDL | % | Relative lipoprotein lipid concentrations |
| S_HDL_CE_pct | Cholesteryl esters to total lipids ratio in small HDL | % | Relative lipoprotein lipid concentrations |
| S_HDL_FC_pct | Free cholesterol to total lipids ratio in small HDL | % | Relative lipoprotein lipid concentrations |
| S_HDL_TG_pct | Triglycerides to total lipids ratio in small HDL | % | Relative lipoprotein lipid concentrations |

**Supplementary Table 2:** **Disease definition code from UK Biobank**

| **Disease code** | **Self_report** | **OPCS-4** | **ICD-9** | **ICD-10** |
| --- | --- | --- | --- | --- |
| Diabetes | 2443(1), 2976, 6153(3), 6177(3), 20002 (1220,1222,1223), 20008 | N.A | 41271(250,3572,3620),41281 | 41270 (E10-E14, G590, G632, H280, H360, M142, N083), 41280 |
| Hypertension | 2966, 6150(4),6 153(2), 6177(2), 20002(1065,1072), 20008 | N.A | 41271(401-405), 41281 | 41270(I10-I13, I15, O10), 41280 |
| Coronary heart disease | 6150(1,2), 3894, 3627, 20004, (1070,1095,1523), 20002(1074,1075), 20008, 20010, | 41272 (K40-K46, K49, K50, K75), 41282 | 41271(410-414),41281 | 41270(I20-I25, Z951, Z955), 41280 |
| Stroke | 6150(3), 4056, 20002, (1081,1491,1583,1086), 20008, 20010 | 41272 (A052-A054, L351, L353, L343), 41282 | 41271(3361,3623,430, 431,4329,4330,4331, 4332,4333,4338,4339, 434,436),41281 | 41270(I60, I61, I629, I63, I64, I678, I690, I693, G951, H341, H342, S066),41280 |

ICD: International Classification of Diseases;

OPCS: Office of Population Censuses and Surveys Classification of Interventions and Procedures;

**Supplementary Table 3:** **Nominally significant associations (P-value < 0.05) between NMR metabolic biomarkers and calcification in the overall population from Model 1.**

| **Metabolic biomarkers** | **Metabolic groups** | **Calcification** | **RS-I** | | | | **RS-II** | | | | **LLS** | | | | **META** | | | |
| --- | --- | --- | --- | --- | --- | --- | --- | --- | --- | --- | --- | --- | --- | --- | --- | --- | --- | --- |
|  |  |  | **Beta** | **SE** | **P** | **N** | **Beta** | **SE** | **P** | **N** | **BETA** | **SE** | **P** | **N** | **Beta** | **SE** | **P** | **N** |
| Glucose | Glycolysis related metabolites | CAC | 0.08 | 0.03 | 1.89E-02 | 671 | 0.15 | 0.05 | 2.08E-03 | 376 | 0.11 | 0.05 | 4.16E-02 | 272 | **0.11** | **0.02** | **2.11E-05** | 1319 |
| L_HDL_CE_pct | Relative lipoprotein lipid concentrations | CAC | -0.09 | 0.04 | 1.21E-02 | 671 | -0.10 | 0.04 | 2.11E-02 | 376 | -0.09 | 0.04 | 3.26E-02 | 272 | **-0.09** | **0.02** | **5.44E-05** | 1319 |
| L_HDL_C_pct | Relative lipoprotein lipid concentrations | CAC | -0.07 | 0.04 | 3.93E-02 | 671 | -0.11 | 0.04 | 8.40E-03 | 376 | -0.09 | 0.04 | 4.72E-02 | 272 | **-0.09** | **0.02** | **1.22E-04** | 1319 |
| Omega_6_pct | Fatty acids | CAC | -0.11 | 0.04 | 3.23E-03 | 671 | -0.07 | 0.05 | 1.59E-01 | 376 | -0.10 | 0.05 | 3.77E-02 | 272 | **-0.10** | **0.02** | **1.23E-04** | 1319 |
| PUFA_by_MUFA | Fatty acids | CAC | -0.09 | 0.04 | 1.06E-02 | 671 | -0.08 | 0.05 | 9.21E-02 | 376 | -0.12 | 0.05 | 1.91E-02 | 272 | **-0.10** | **0.02** | **1.26E-04** | 1319 |
| GlycA | Inflammation | AAC | 0.10 | 0.03 | 3.47E-03 | 671 | 0.14 | 0.05 | 1.19E-02 | 376 | NA | NA | NA | NA | **0.11** | **0.03** | **1.27E-04** | 1047 |
| L_LDL_TG_pct | Relative lipoprotein lipid concentrations | CAC | 0.07 | 0.04 | 4.59E-02 | 671 | 0.14 | 0.05 | 2.80E-03 | 376 | 0.08 | 0.05 | 1.13E-01 | 272 | **0.10** | **0.03** | **1.65E-04** | 1319 |
| LA_pct | Fatty acids | CAC | -0.07 | 0.04 | 3.48E-02 | 671 | -0.12 | 0.05 | 1.41E-02 | 376 | -0.10 | 0.05 | 4.33E-02 | 272 | **-0.09** | **0.02** | **1.77E-04** | 1319 |
| PUFA_pct | Fatty acids | CAC | -0.09 | 0.04 | 9.74E-03 | 671 | -0.07 | 0.05 | 1.70E-01 | 376 | -0.12 | 0.05 | 1.56E-02 | 272 | **-0.09** | **0.02** | **1.77E-04** | 1319 |
| XS_VLDL_CE_pct | Relative lipoprotein lipid concentrations | CAC | -0.06 | 0.04 | 1.38E-01 | 671 | -0.14 | 0.04 | 1.88E-03 | 376 | -0.10 | 0.05 | 2.73E-02 | 272 | **-0.09** | **0.03** | **2.50E-04** | 1319 |
| MUFA_pct | Fatty acids | CAC | 0.08 | 0.03 | 2.05E-02 | 671 | 0.09 | 0.05 | 7.42E-02 | 376 | 0.11 | 0.05 | 3.57E-02 | 272 | **0.09** | **0.02** | **3.38E-04** | 1319 |
| IDL_TG_pct | Relative lipoprotein lipid concentrations | CAC | 0.06 | 0.04 | 8.79E-02 | 671 | 0.14 | 0.05 | 4.28E-03 | 376 | 0.09 | 0.05 | 9.44E-02 | 272 | **0.09** | **0.03** | **4.01E-04** | 1319 |
| M_LDL_TG_pct | Relative lipoprotein lipid concentrations | CAC | 0.07 | 0.04 | 5.09E-02 | 671 | 0.12 | 0.05 | 1.04E-02 | 376 | 0.09 | 0.05 | 9.86E-02 | 272 | **0.09** | **0.03** | **4.05E-04** | 1319 |
| Citrate | Glycolysis related metabolites | CAC | 0.08 | 0.04 | 2.75E-02 | 671 | 0.06 | 0.04 | 1.72E-01 | 376 | 0.14 | 0.05 | 7.88E-03 | 272 | **0.09** | **0.02** | **4.19E-04** | 1319 |
| M_LDL_FC_pct | Relative lipoprotein lipid concentrations | CAC | -0.07 | 0.04 | 5.11E-02 | 671 | -0.11 | 0.05 | 3.53E-02 | 376 | -0.10 | 0.05 | 4.76E-02 | 272 | -0.09 | 0.03 | 5.78E-04 | 1319 |
| S_LDL_TG_pct | Relative lipoprotein lipid concentrations | CAC | 0.08 | 0.04 | 3.87E-02 | 671 | 0.11 | 0.05 | 2.91E-02 | 376 | 0.08 | 0.05 | 1.13E-01 | 272 | 0.09 | 0.03 | 7.69E-04 | 1319 |
| S_LDL_FC_pct | Relative lipoprotein lipid concentrations | CAC | -0.07 | 0.04 | 6.20E-02 | 671 | -0.09 | 0.05 | 6.01E-02 | 376 | -0.10 | 0.05 | 4.54E-02 | 272 | -0.08 | 0.02 | 1.05E-03 | 1319 |
| L_LDL_FC_pct | Relative lipoprotein lipid concentrations | CAC | -0.07 | 0.04 | 6.59E-02 | 671 | -0.09 | 0.05 | 6.47E-02 | 376 | -0.10 | 0.05 | 4.21E-02 | 272 | -0.08 | 0.02 | 1.07E-03 | 1319 |
| XS_VLDL_C_pct | Relative lipoprotein lipid concentrations | CAC | -0.05 | 0.04 | 1.79E-01 | 671 | -0.15 | 0.04 | 1.06E-03 | 376 | -0.11 | 0.05 | 2.27E-02 | 272 | -0.10 | 0.03 | 1.30E-03 | 1319 |
| L_HDL_CE | Lipoprotein subclasses | CAC | -0.06 | 0.04 | 1.28E-01 | 671 | -0.10 | 0.05 | 3.69E-02 | 376 | -0.10 | 0.05 | 4.02E-02 | 272 | -0.08 | 0.02 | 1.43E-03 | 1319 |
| M_HDL_CE_pct | Relative lipoprotein lipid concentrations | CAC | -0.09 | 0.04 | 1.59E-02 | 671 | -0.07 | 0.05 | 1.54E-01 | 376 | -0.07 | 0.05 | 1.32E-01 | 272 | -0.08 | 0.03 | 1.57E-03 | 1319 |
| GlycA | Inflammation | CAC | 0.09 | 0.04 | 1.58E-02 | 671 | 0.05 | 0.05 | 2.79E-01 | 376 | 0.09 | 0.05 | 7.97E-02 | 272 | 0.08 | 0.03 | 1.81E-03 | 1319 |
| XL_HDL_TG_pct | Relative lipoprotein lipid concentrations | CAC | 0.08 | 0.04 | 2.47E-02 | 671 | 0.07 | 0.05 | 1.30E-01 | 376 | 0.07 | 0.05 | 1.51E-01 | 272 | 0.08 | 0.02 | 2.16E-03 | 1319 |
| M_HDL_C_pct | Relative lipoprotein lipid concentrations | CAC | -0.08 | 0.04 | 4.08E-02 | 671 | -0.08 | 0.05 | 8.66E-02 | 376 | -0.07 | 0.05 | 1.57E-01 | 272 | -0.08 | 0.03 | 2.48E-03 | 1319 |
| L_HDL_C | Lipoprotein subclasses | CAC | -0.05 | 0.04 | 1.64E-01 | 671 | -0.10 | 0.05 | 3.15E-02 | 376 | -0.09 | 0.05 | 6.97E-02 | 272 | -0.08 | 0.03 | 2.63E-03 | 1319 |
| IDL_FC_pct | Relative lipoprotein lipid concentrations | CAC | -0.05 | 0.04 | 1.88E-01 | 671 | -0.12 | 0.04 | 6.66E-03 | 376 | -0.06 | 0.05 | 2.22E-01 | 272 | -0.07 | 0.02 | 2.63E-03 | 1319 |
| L_LDL_C_pct | Relative lipoprotein lipid concentrations | CAC | -0.06 | 0.04 | 1.12E-01 | 671 | -0.15 | 0.04 | 6.20E-04 | 376 | -0.07 | 0.05 | 1.84E-01 | 272 | -0.09 | 0.03 | 2.76E-03 | 1319 |
| S_LDL_C_pct | Relative lipoprotein lipid concentrations | CAC | -0.05 | 0.04 | 1.69E-01 | 671 | -0.09 | 0.05 | 5.92E-02 | 376 | -0.10 | 0.05 | 4.09E-02 | 272 | -0.07 | 0.02 | 2.94E-03 | 1319 |
| IDL_C_pct | Relative lipoprotein lipid concentrations | CAC | -0.05 | 0.04 | 1.49E-01 | 671 | -0.09 | 0.05 | 4.30E-02 | 376 | -0.09 | 0.05 | 7.90E-02 | 272 | -0.07 | 0.03 | 3.20E-03 | 1319 |
| LDL_size | Lipoprotein particle sizes | CAC | -0.06 | 0.04 | 9.22E-02 | 671 | -0.08 | 0.05 | 7.66E-02 | 376 | -0.08 | 0.05 | 1.08E-01 | 272 | -0.07 | 0.02 | 3.76E-03 | 1319 |
| M_VLDL_CE_pct | Relative lipoprotein lipid concentrations | CAC | -0.04 | 0.04 | 2.19E-01 | 671 | -0.09 | 0.05 | 4.10E-02 | 376 | -0.08 | 0.05 | 8.07E-02 | 272 | -0.07 | 0.02 | 4.78E-03 | 1319 |
| S_HDL_CE_pct | Relative lipoprotein lipid concentrations | CAC | -0.07 | 0.04 | 5.17E-02 | 671 | -0.07 | 0.05 | 1.62E-01 | 376 | -0.07 | 0.05 | 1.59E-01 | 272 | -0.07 | 0.03 | 5.39E-03 | 1319 |
| L_HDL_PL_pct | Relative lipoprotein lipid concentrations | CAC | 0.04 | 0.04 | 2.45E-01 | 671 | 0.13 | 0.05 | 4.53E-03 | 376 | 0.09 | 0.05 | 8.73E-02 | 272 | 0.08 | 0.03 | 5.76E-03 | 1319 |
| L_HDL_TG_pct | Relative lipoprotein lipid concentrations | CAC | 0.07 | 0.04 | 4.70E-02 | 671 | 0.08 | 0.05 | 7.60E-02 | 376 | 0.04 | 0.05 | 4.34E-01 | 272 | 0.07 | 0.03 | 6.72E-03 | 1319 |
| S_VLDL_PL_pct | Relative lipoprotein lipid concentrations | CAC | -0.04 | 0.04 | 2.63E-01 | 671 | -0.13 | 0.05 | 1.02E-02 | 376 | -0.07 | 0.05 | 1.80E-01 | 272 | -0.07 | 0.03 | 6.94E-03 | 1319 |
| S_HDL_TG_pct | Relative lipoprotein lipid concentrations | CAC | 0.08 | 0.04 | 4.29E-02 | 671 | 0.07 | 0.05 | 1.62E-01 | 376 | 0.06 | 0.05 | 2.85E-01 | 272 | 0.07 | 0.03 | 7.46E-03 | 1319 |
| M_HDL_PL_pct | Relative lipoprotein lipid concentrations | CAC | 0.06 | 0.04 | 1.16E-01 | 671 | 0.07 | 0.05 | 1.28E-01 | 376 | 0.08 | 0.05 | 1.24E-01 | 272 | 0.07 | 0.02 | 7.82E-03 | 1319 |
| L_HDL_FC | Lipoprotein subclasses | CAC | -0.03 | 0.04 | 3.57E-01 | 671 | -0.11 | 0.05 | 2.07E-02 | 376 | -0.08 | 0.05 | 1.21E-01 | 272 | -0.07 | 0.02 | 8.27E-03 | 1319 |
| S_HDL_C_pct | Relative lipoprotein lipid concentrations | CAC | -0.05 | 0.04 | 1.30E-01 | 671 | -0.09 | 0.05 | 5.20E-02 | 376 | -0.06 | 0.05 | 2.65E-01 | 272 | -0.07 | 0.02 | 8.28E-03 | 1319 |
| XS_VLDL_TG_pct | Relative lipoprotein lipid concentrations | CAC | 0.04 | 0.04 | 3.52E-01 | 671 | 0.13 | 0.05 | 6.37E-03 | 376 | 0.09 | 0.05 | 1.08E-01 | 272 | 0.08 | 0.03 | 8.89E-03 | 1319 |
| S_VLDL_FC_pct | Relative lipoprotein lipid concentrations | CAC | -0.04 | 0.04 | 3.08E-01 | 671 | -0.13 | 0.05 | 6.89E-03 | 376 | -0.09 | 0.05 | 1.09E-01 | 272 | -0.08 | 0.03 | 9.02E-03 | 1319 |
| IDL_TG | Lipoprotein subclasses | AVC | 0.11 | 0.05 | 1.41E-02 | 671 | 0.05 | 0.05 | 2.49E-01 | 376 | NA | NA | NA | NA | 0.08 | 0.03 | 1.06E-02 | 1047 |
| XXL_VLDL_L | Lipoprotein subclasses | CAC | 0.09 | 0.04 | 1.39E-02 | 671 | 0.02 | 0.04 | 6.54E-01 | 376 | 0.07 | 0.05 | 2.15E-01 | 272 | 0.06 | 0.02 | 1.11E-02 | 1319 |
| Ala | Amino acids | CAC | 0.04 | 0.03 | 2.31E-01 | 671 | 0.07 | 0.05 | 1.37E-01 | 376 | 0.10 | 0.05 | 5.59E-02 | 272 | 0.06 | 0.02 | 1.11E-02 | 1319 |
| M_VLDL_C_pct | Relative lipoprotein lipid concentrations | CAC | -0.04 | 0.04 | 2.98E-01 | 671 | -0.09 | 0.05 | 4.59E-02 | 376 | -0.08 | 0.05 | 1.27E-01 | 272 | -0.06 | 0.02 | 1.15E-02 | 1319 |
| XXL_VLDL_FC | Lipoprotein subclasses | CAC | 0.09 | 0.04 | 1.78E-02 | 671 | 0.02 | 0.05 | 6.80E-01 | 376 | 0.07 | 0.05 | 1.76E-01 | 272 | 0.06 | 0.03 | 1.21E-02 | 1319 |
| L_HDL_P | Lipoprotein subclasses | CAC | -0.04 | 0.04 | 3.03E-01 | 671 | -0.10 | 0.05 | 3.91E-02 | 376 | -0.08 | 0.06 | 1.59E-01 | 272 | -0.06 | 0.03 | 1.24E-02 | 1319 |
| XXL_VLDL_P | Lipoprotein subclasses | CAC | 0.09 | 0.04 | 1.03E-02 | 671 | 0.02 | 0.04 | 7.04E-01 | 376 | 0.06 | 0.05 | 2.31E-01 | 272 | 0.06 | 0.03 | 1.32E-02 | 1319 |
| TG_by_PG | Other lipids | CAC | 0.08 | 0.04 | 3.99E-02 | 671 | 0.03 | 0.05 | 4.65E-01 | 376 | 0.07 | 0.05 | 1.77E-01 | 272 | 0.06 | 0.03 | 1.35E-02 | 1319 |
| S_HDL_TG | Lipoprotein subclasses | CAC | 0.08 | 0.04 | 3.37E-02 | 671 | 0.06 | 0.05 | 2.68E-01 | 376 | 0.04 | 0.05 | 4.27E-01 | 272 | 0.06 | 0.03 | 1.41E-02 | 1319 |
| XXL_VLDL_C | Lipoprotein subclasses | CAC | 0.08 | 0.04 | 1.90E-02 | 671 | 0.01 | 0.05 | 8.79E-01 | 376 | 0.08 | 0.05 | 1.54E-01 | 272 | 0.06 | 0.03 | 1.56E-02 | 1319 |
| L_HDL_L | Lipoprotein subclasses | CAC | -0.04 | 0.04 | 2.46E-01 | 671 | -0.09 | 0.05 | 5.71E-02 | 376 | -0.07 | 0.06 | 2.28E-01 | 272 | -0.06 | 0.03 | 1.60E-02 | 1319 |
| SFA_pct | Fatty acids | AVC | 0.05 | 0.04 | 2.68E-01 | 671 | 0.10 | 0.04 | 2.06E-02 | 376 | NA | NA | NA | NA | 0.07 | 0.03 | 1.63E-02 | 1047 |
| HDL_CE | Cholesteryl esters | CAC | -0.04 | 0.04 | 2.58E-01 | 671 | -0.08 | 0.05 | 7.68E-02 | 376 | -0.07 | 0.06 | 2.05E-01 | 272 | -0.06 | 0.03 | 1.85E-02 | 1319 |
| Gly | Amino acids | CAC | -0.09 | 0.04 | 1.10E-02 | 671 | -0.04 | 0.05 | 4.70E-01 | 376 | -0.01 | 0.06 | 8.01E-01 | 272 | -0.06 | 0.03 | 1.92E-02 | 1319 |
| Ile | Amino acids | CAC | 0.05 | 0.04 | 1.89E-01 | 671 | 0.08 | 0.05 | 1.60E-01 | 376 | 0.09 | 0.06 | 1.66E-01 | 272 | 0.07 | 0.03 | 2.05E-02 | 1319 |
| XL_HDL_FC_pct | Relative lipoprotein lipid concentrations | CAC | 0.04 | 0.03 | 3.08E-01 | 671 | 0.09 | 0.05 | 6.03E-02 | 376 | 0.07 | 0.05 | 1.94E-01 | 272 | 0.06 | 0.02 | 2.09E-02 | 1319 |
| LDL_TG | Triglycerides | AVC | 0.11 | 0.05 | 2.02E-02 | 671 | 0.04 | 0.05 | 3.58E-01 | 376 | NA | NA | NA | NA | 0.07 | 0.03 | 2.25E-02 | 1047 |
| XXL_VLDL_CE_pct | Relative lipoprotein lipid concentrations | CAC | -0.08 | 0.04 | 2.43E-02 | 671 | -0.03 | 0.04 | 3.88E-01 | 376 | NA | NA | NA | NA | -0.06 | 0.03 | 2.34E-02 | 1047 |
| XS_VLDL_TG | Lipoprotein subclasses | CAC | 0.11 | 0.04 | 5.27E-03 | 671 | 0.01 | 0.05 | 8.19E-01 | 376 | 0.06 | 0.05 | 2.36E-01 | 272 | 0.07 | 0.03 | 2.39E-02 | 1319 |
| XS_VLDL_PL_pct | Relative lipoprotein lipid concentrations | CAC | 0.07 | 0.04 | 6.61E-02 | 671 | 0.04 | 0.04 | 2.95E-01 | 376 | 0.05 | 0.05 | 3.35E-01 | 272 | 0.05 | 0.02 | 2.47E-02 | 1319 |
| Omega_6_pct | Fatty acids | AAC | -0.05 | 0.03 | 1.65E-01 | 671 | -0.11 | 0.06 | 4.55E-02 | 376 | NA | NA | NA | NA | -0.07 | 0.03 | 2.52E-02 | 1047 |
| M_HDL_TG_pct | Relative lipoprotein lipid concentrations | CAC | 0.07 | 0.04 | 7.40E-02 | 671 | 0.06 | 0.05 | 2.12E-01 | 376 | 0.04 | 0.05 | 5.01E-01 | 272 | 0.06 | 0.03 | 2.54E-02 | 1319 |
| S_LDL_C_pct | Relative lipoprotein lipid concentrations | AAC | -0.05 | 0.03 | 1.16E-01 | 671 | -0.13 | 0.05 | 8.76E-03 | 376 | NA | NA | NA | NA | -0.09 | 0.04 | 2.63E-02 | 1047 |
| XXL_VLDL_C_pct | Relative lipoprotein lipid concentrations | CAC | -0.08 | 0.04 | 2.10E-02 | 671 | -0.03 | 0.04 | 5.02E-01 | 376 | NA | NA | NA | NA | -0.06 | 0.03 | 2.73E-02 | 1047 |
| Unsaturation | Fatty acids | CAC | -0.03 | 0.03 | 3.49E-01 | 671 | -0.06 | 0.05 | 2.25E-01 | 376 | -0.10 | 0.05 | 5.83E-02 | 272 | -0.06 | 0.03 | 2.75E-02 | 1319 |
| L_HDL_PL | Lipoprotein subclasses | CAC | -0.04 | 0.04 | 2.70E-01 | 671 | -0.08 | 0.05 | 8.68E-02 | 376 | -0.06 | 0.06 | 2.84E-01 | 272 | -0.06 | 0.03 | 2.77E-02 | 1319 |
| HDL_C | Cholesterol | CAC | -0.04 | 0.04 | 3.49E-01 | 671 | -0.09 | 0.05 | 6.65E-02 | 376 | -0.07 | 0.06 | 2.53E-01 | 272 | -0.06 | 0.03 | 2.79E-02 | 1319 |
| XXL_VLDL_PL | Lipoprotein subclasses | CAC | 0.07 | 0.04 | 4.91E-02 | 671 | 0.02 | 0.05 | 6.06E-01 | 376 | 0.06 | 0.05 | 2.47E-01 | 272 | 0.06 | 0.03 | 2.80E-02 | 1319 |
| XL_HDL_PL | Lipoprotein subclasses | CAC | -0.02 | 0.03 | 5.35E-01 | 671 | -0.08 | 0.04 | 6.37E-02 | 376 | -0.08 | 0.05 | 1.16E-01 | 272 | -0.05 | 0.02 | 2.81E-02 | 1319 |
| S_VLDL_C_pct | Relative lipoprotein lipid concentrations | CAC | -0.02 | 0.04 | 5.50E-01 | 671 | -0.09 | 0.05 | 5.94E-02 | 376 | -0.08 | 0.05 | 1.28E-01 | 272 | -0.05 | 0.02 | 2.85E-02 | 1319 |
| XS_VLDL_TG | Lipoprotein subclasses | AVC | 0.08 | 0.05 | 7.17E-02 | 671 | 0.06 | 0.04 | 1.96E-01 | 376 | NA | NA | NA | NA | 0.07 | 0.03 | 2.89E-02 | 1047 |
| L_LDL_TG_pct | Relative lipoprotein lipid concentrations | AVC | 0.05 | 0.04 | 2.22E-01 | 671 | 0.08 | 0.04 | 7.01E-02 | 376 | NA | NA | NA | NA | 0.07 | 0.03 | 3.10E-02 | 1047 |
| M_LDL_C_pct | Relative lipoprotein lipid concentrations | CAC | -0.02 | 0.04 | 5.42E-01 | 671 | -0.08 | 0.04 | 7.12E-02 | 376 | -0.08 | 0.05 | 1.50E-01 | 272 | -0.05 | 0.02 | 3.57E-02 | 1319 |
| Val | Amino acids | CAC | 0.08 | 0.04 | 4.50E-02 | 671 | 0.04 | 0.05 | 4.28E-01 | 376 | 0.03 | 0.06 | 5.77E-01 | 272 | 0.06 | 0.03 | 3.58E-02 | 1319 |
| Total_BCAA | Amino acids | CAC | 0.07 | 0.04 | 9.15E-02 | 671 | 0.06 | 0.05 | 2.91E-01 | 376 | 0.04 | 0.06 | 4.82E-01 | 272 | 0.06 | 0.03 | 3.72E-02 | 1319 |
| LA_pct | Fatty acids | AAC | -0.06 | 0.03 | 8.22E-02 | 671 | -0.16 | 0.05 | 4.46E-03 | 376 | NA | NA | NA | NA | -0.10 | 0.05 | 3.77E-02 | 1047 |
| XL_HDL_CE | Lipoprotein subclasses | CAC | -0.03 | 0.04 | 4.89E-01 | 671 | -0.13 | 0.05 | 5.30E-03 | 376 | -0.07 | 0.05 | 2.31E-01 | 272 | -0.07 | 0.03 | 3.90E-02 | 1319 |
| HDL_TG | Triglycerides | AVC | 0.05 | 0.05 | 2.93E-01 | 671 | 0.09 | 0.05 | 6.31E-02 | 376 | NA | NA | NA | NA | 0.07 | 0.03 | 3.92E-02 | 1047 |
| HDL_TG | Triglycerides | CAC | 0.08 | 0.04 | 4.35E-02 | 671 | 0.04 | 0.05 | 4.26E-01 | 376 | 0.03 | 0.05 | 6.31E-01 | 272 | 0.05 | 0.03 | 3.97E-02 | 1319 |
| IDL_CE_pct | Relative lipoprotein lipid concentrations | CAC | -0.04 | 0.04 | 2.94E-01 | 671 | -0.05 | 0.04 | 2.92E-01 | 376 | -0.08 | 0.05 | 1.17E-01 | 272 | -0.05 | 0.03 | 4.03E-02 | 1319 |
| L_LDL_TG | Lipoprotein subclasses | AVC | 0.12 | 0.05 | 9.22E-03 | 671 | 0.04 | 0.05 | 3.69E-01 | 376 | NA | NA | NA | NA | 0.08 | 0.04 | 4.10E-02 | 1047 |
| L_LDL_C_pct | Relative lipoprotein lipid concentrations | AVC | -0.04 | 0.04 | 4.10E-01 | 671 | -0.08 | 0.04 | 4.56E-02 | 376 | NA | NA | NA | NA | -0.06 | 0.03 | 4.14E-02 | 1047 |
| PUFA_pct | Fatty acids | AAC | -0.05 | 0.03 | 1.60E-01 | 671 | -0.09 | 0.06 | 1.09E-01 | 376 | NA | NA | NA | NA | -0.06 | 0.03 | 4.22E-02 | 1047 |
| M_LDL_TG | Lipoprotein subclasses | AVC | 0.09 | 0.05 | 4.10E-02 | 671 | 0.04 | 0.04 | 4.02E-01 | 376 | NA | NA | NA | NA | 0.06 | 0.03 | 4.23E-02 | 1047 |
| Pyruvate | Glycolysis related metabolites | CAC | 0.05 | 0.04 | 1.41E-01 | 671 | 0.04 | 0.04 | 2.83E-01 | 376 | 0.05 | 0.05 | 3.67E-01 | 272 | 0.05 | 0.02 | 4.26E-02 | 1319 |
| M_HDL_CE | Lipoprotein subclasses | CAC | -0.04 | 0.04 | 2.68E-01 | 671 | -0.05 | 0.05 | 2.63E-01 | 376 | -0.08 | 0.06 | 1.89E-01 | 272 | -0.05 | 0.03 | 4.59E-02 | 1319 |
| L_LDL_TG | Lipoprotein subclasses | AAC | 0.05 | 0.04 | 2.25E-01 | 671 | 0.10 | 0.06 | 7.59E-02 | 376 | NA | NA | NA | NA | 0.06 | 0.03 | 4.70E-02 | 1047 |
| S_HDL_CE_pct | Relative lipoprotein lipid concentrations | AVC | -0.04 | 0.04 | 3.73E-01 | 671 | -0.08 | 0.04 | 6.14E-02 | 376 | NA | NA | NA | NA | -0.06 | 0.03 | 4.79E-02 | 1047 |
| M_HDL_TG | Lipoprotein subclasses | AVC | 0.04 | 0.05 | 4.15E-01 | 671 | 0.09 | 0.05 | 4.91E-02 | 376 | NA | NA | NA | NA | 0.06 | 0.03 | 4.82E-02 | 1047 |
| S_VLDL_TG | Lipoprotein subclasses | CAC | 0.08 | 0.04 | 2.50E-02 | 671 | 0.00 | 0.05 | 9.71E-01 | 376 | 0.06 | 0.05 | 2.52E-01 | 272 | 0.05 | 0.03 | 4.96E-02 | 1319 |

Bold indicates significant association from multi-test correction (P-value < 4.39×10⁻⁴). Model 1 was adjusted for age, sex, and lipid-lowering medication.

Metabolic biomarkers: the biomarkers used as exposures.

Metabolic group: the NMR biomarker subgroups.

Calcification: site-specific calcification used as the outcome, CAC: coronary artery calcification; AAC: aortic arch calcification; AVC: aortic valve calcification.

Beta: estimated standard deviation changes of calcification across one standard deviation increase of metabolic biomarkers.

SE: standard error of the estimated effect size.

P-value: strength of evidence against the null hypothesis of no association between biomarker and calcification.

N: number of participants included in the analysis.

**Supplementary Table 4:** **Nominally significant association (P-value < 0.05) between NMR metabolic biomarkers and calcification in the** **overall population from model2.**

| **Metabolic biomarkers** | **Metabolic group** | **Calcification** | **RS-I** | | | | **RS-II** | | | | **LLS** | | | | **Meta** | | | |
| --- | --- | --- | --- | --- | --- | --- | --- | --- | --- | --- | --- | --- | --- | --- | --- | --- | --- | --- |
|  |  |  | **Beta** | **SE** | **P** | **N** | **Beta** | **SE** | **P** | **N** | **Beta** | **SE** | **P** | **N** | **Beta** | **SE** | **P** | **N** |
| Omega_6_pct | Fatty acids | CAC | -0.11 | 0.04 | 3.66E-03 | 643 | -0.04 | 0.05 | 4.41E-01 | 370 | -0.11 | 0.06 | 6.15E-02 | 210 | **-0.10** | **0.03** | **2.94E-04** | 1214 |
| PUFA_by_MUFA | Fatty acids | CAC | -0.09 | 0.04 | 1.15E-02 | 643 | -0.06 | 0.05 | 2.86E-01 | 370 | -0.13 | 0.06 | 2.53E-02 | 210 | **-0.10** | **0.03** | **3.08E-04** | 1214 |
| PUFA_pct | Fatty acids | CAC | -0.10 | 0.04 | 7.60E-03 | 643 | -0.04 | 0.05 | 4.21E-01 | 370 | -0.12 | 0.06 | 3.38E-02 | 210 | **-0.10** | **0.03** | **3.33E-04** | 1214 |
| LA_pct | Fatty acids | CAC | -0.08 | 0.04 | 3.11E-02 | 643 | -0.08 | 0.05 | 1.14E-01 | 370 | -0.11 | 0.06 | 7.13E-02 | 210 | -0.09 | 0.03 | 6.62E-04 | 1214 |
| L_HDL_CE_pct | Relative lipoprotein lipid concentrations | CAC | -0.08 | 0.04 | 4.04E-02 | 643 | -0.09 | 0.05 | 4.31E-02 | 370 | -0.09 | 0.06 | 1.23E-01 | 210 | -0.09 | 0.03 | 7.58E-04 | 1214 |
| L_HDL_C_pct | Relative lipoprotein lipid concentrations | CAC | -0.06 | 0.04 | 1.00E-01 | 643 | -0.11 | 0.05 | 1.53E-02 | 370 | -0.15 | 0.09 | 9.78E-02 | 210 | -0.09 | 0.03 | 9.54E-04 | 1214 |
| MUFA_pct | Fatty acids | CAC | 0.08 | 0.04 | 3.32E-02 | 643 | 0.06 | 0.05 | 2.43E-01 | 370 | 0.13 | 0.06 | 3.20E-02 | 210 | 0.09 | 0.03 | 1.15E-03 | 1214 |
| M_LDL_FC_pct | Relative lipoprotein lipid concentrations | CAC | -0.07 | 0.04 | 4.33E-02 | 643 | -0.07 | 0.05 | 1.61E-01 | 370 | -0.10 | 0.06 | 8.15E-02 | 210 | -0.08 | 0.03 | 1.40E-03 | 1214 |
| XS_VLDL_CE_pct | Relative lipoprotein lipid concentrations | CAC | -0.05 | 0.04 | 2.19E-01 | 643 | -0.10 | 0.05 | 3.18E-02 | 370 | -0.09 | 0.05 | 5.48E-02 | 210 | -0.08 | 0.03 | 1.91E-03 | 1214 |
| S_LDL_FC_pct | Relative lipoprotein lipid concentrations | CAC | -0.07 | 0.04 | 6.05E-02 | 643 | -0.08 | 0.05 | 1.34E-01 | 370 | -0.09 | 0.05 | 1.08E-01 | 210 | -0.08 | 0.03 | 2.09E-03 | 1214 |
| L_LDL_TG_pct | Relative lipoprotein lipid concentrations | CAC | 0.07 | 0.04 | 6.42E-02 | 643 | 0.09 | 0.05 | 7.01E-02 | 370 | 0.06 | 0.06 | 2.96E-01 | 210 | 0.08 | 0.03 | 2.58E-03 | 1214 |
| L_LDL_C_pct | Relative lipoprotein lipid concentrations | CAC | -0.06 | 0.04 | 1.10E-01 | 643 | -0.11 | 0.05 | 1.88E-02 | 370 | -0.05 | 0.06 | 4.17E-01 | 210 | -0.08 | 0.03 | 2.79E-03 | 1214 |
| XS_VLDL_C_pct | Relative lipoprotein lipid concentrations | CAC | -0.04 | 0.04 | 2.76E-01 | 643 | -0.11 | 0.05 | 2.37E-02 | 370 | -0.11 | 0.05 | 3.45E-02 | 210 | -0.09 | 0.03 | 2.80E-03 | 1214 |
| S_LDL_TG_pct | Relative lipoprotein lipid concentrations | CAC | 0.08 | 0.04 | 2.95E-02 | 643 | 0.07 | 0.05 | 2.18E-01 | 370 | 0.07 | 0.06 | 2.17E-01 | 210 | 0.08 | 0.03 | 3.03E-03 | 1214 |
| M_HDL_CE_pct | Relative lipoprotein lipid concentrations | CAC | -0.08 | 0.04 | 3.58E-02 | 643 | -0.05 | 0.05 | 3.24E-01 | 370 | -0.08 | 0.06 | 1.65E-01 | 210 | -0.08 | 0.03 | 3.42E-03 | 1214 |
| LDL_size | Lipoprotein particle sizes | CAC | -0.07 | 0.04 | 3.82E-02 | 643 | -0.05 | 0.05 | 2.54E-01 | 370 | -0.09 | 0.06 | 1.30E-01 | 210 | -0.07 | 0.03 | 3.90E-03 | 1214 |
| Glucose | Glycolysis related metabolites | CAC | 0.07 | 0.05 | 1.51E-01 | 643 | 0.11 | 0.05 | 4.20E-02 | 370 | 0.10 | 0.07 | 1.40E-01 | 210 | 0.09 | 0.03 | 4.04E-03 | 1214 |
| GlycA | Inflammation | AAC | 0.10 | 0.04 | 8.03E-03 | 643 | 0.07 | 0.06 | 2.44E-01 | 370 | NA | NA | NA | NA | 0.09 | 0.03 | 4.19E-03 | 1013 |
| M_HDL_C_pct | Relative lipoprotein lipid concentrations | CAC | -0.07 | 0.04 | 6.50E-02 | 643 | -0.06 | 0.05 | 2.28E-01 | 370 | -0.08 | 0.06 | 1.69E-01 | 210 | -0.08 | 0.03 | 4.41E-03 | 1214 |
| M_LDL_TG_pct | Relative lipoprotein lipid concentrations | CAC | 0.07 | 0.04 | 6.15E-02 | 643 | 0.07 | 0.05 | 1.49E-01 | 370 | 0.07 | 0.06 | 2.66E-01 | 210 | 0.08 | 0.03 | 4.56E-03 | 1214 |
| IDL_TG_pct | Relative lipoprotein lipid concentrations | CAC | 0.06 | 0.04 | 1.26E-01 | 643 | 0.09 | 0.05 | 8.70E-02 | 370 | 0.08 | 0.06 | 1.91E-01 | 210 | 0.08 | 0.03 | 4.76E-03 | 1214 |
| L_LDL_FC_pct | Relative lipoprotein lipid concentrations | CAC | -0.07 | 0.04 | 8.55E-02 | 643 | -0.05 | 0.05 | 3.08E-01 | 370 | -0.10 | 0.05 | 5.53E-02 | 210 | -0.07 | 0.03 | 5.00E-03 | 1214 |
| Citrate | Glycolysis related metabolites | CAC | 0.06 | 0.04 | 6.87E-02 | 643 | 0.07 | 0.05 | 1.41E-01 | 370 | 0.10 | 0.06 | 1.03E-01 | 210 | 0.07 | 0.03 | 5.91E-03 | 1214 |
| L_HDL_CE | Lipoprotein subclasses | CAC | -0.05 | 0.04 | 2.09E-01 | 643 | -0.08 | 0.05 | 9.71E-02 | 370 | -0.12 | 0.06 | 6.76E-02 | 210 | -0.08 | 0.03 | 5.97E-03 | 1214 |
| XL_HDL_TG_pct | Relative lipoprotein lipid concentrations | CAC | 0.07 | 0.04 | 6.82E-02 | 643 | 0.05 | 0.05 | 2.63E-01 | 370 | 0.09 | 0.06 | 1.41E-01 | 210 | 0.07 | 0.03 | 5.99E-03 | 1214 |
| GlycA | Inflammation | CAC | 0.08 | 0.04 | 3.71E-02 | 643 | 0.02 | 0.05 | 6.95E-01 | 370 | 0.11 | 0.06 | 7.41E-02 | 210 | 0.07 | 0.03 | 6.36E-03 | 1214 |
| SFA_pct | Fatty acids | AVC | 0.05 | 0.04 | 2.07E-01 | 643 | 0.11 | 0.04 | 1.13E-02 | 370 | NA | NA | NA | NA | 0.08 | 0.03 | 7.73E-03 | 1013 |
| L_HDL_C | Lipoprotein subclasses | CAC | -0.04 | 0.04 | 2.58E-01 | 643 | -0.09 | 0.05 | 8.46E-02 | 370 | -0.12 | 0.07 | 8.86E-02 | 210 | -0.07 | 0.03 | 8.99E-03 | 1214 |
| L_HDL_TG_pct | Relative lipoprotein lipid concentrations | CAC | 0.07 | 0.04 | 7.38E-02 | 643 | 0.07 | 0.05 | 1.60E-01 | 370 | 0.04 | 0.06 | 5.30E-01 | 210 | 0.07 | 0.03 | 9.43E-03 | 1214 |
| S_HDL_C_pct | Relative lipoprotein lipid concentrations | CAC | -0.06 | 0.04 | 7.37E-02 | 643 | -0.06 | 0.05 | 1.72E-01 | 370 | -0.05 | 0.06 | 4.30E-01 | 210 | -0.07 | 0.03 | 9.48E-03 | 1214 |
| IDL_FC_pct | Relative lipoprotein lipid concentrations | CAC | -0.03 | 0.04 | 3.96E-01 | 643 | -0.10 | 0.04 | 2.76E-02 | 370 | -0.07 | 0.05 | 1.78E-01 | 210 | -0.06 | 0.02 | 9.85E-03 | 1214 |
| S_VLDL_FC_pct | Relative lipoprotein lipid concentrations | CAC | -0.05 | 0.04 | 1.96E-01 | 643 | -0.08 | 0.05 | 1.25E-01 | 370 | -0.10 | 0.06 | 1.17E-01 | 210 | -0.07 | 0.03 | 9.92E-03 | 1214 |
| S_HDL_CE_pct | Relative lipoprotein lipid concentrations | CAC | -0.07 | 0.04 | 4.64E-02 | 643 | -0.04 | 0.05 | 3.70E-01 | 370 | -0.05 | 0.06 | 3.71E-01 | 210 | -0.07 | 0.03 | 1.07E-02 | 1214 |
| M_HDL_PL_pct | Relative lipoprotein lipid concentrations | CAC | 0.06 | 0.04 | 1.34E-01 | 643 | 0.04 | 0.05 | 3.82E-01 | 370 | 0.10 | 0.06 | 9.59E-02 | 210 | 0.07 | 0.03 | 1.25E-02 | 1214 |
| S_VLDL_PL_pct | Relative lipoprotein lipid concentrations | CAC | -0.06 | 0.04 | 1.48E-01 | 643 | -0.07 | 0.05 | 1.64E-01 | 370 | -0.08 | 0.06 | 2.23E-01 | 210 | -0.07 | 0.03 | 1.38E-02 | 1214 |
| XS_VLDL_TG_pct | Relative lipoprotein lipid concentrations | CAC | 0.04 | 0.04 | 3.53E-01 | 643 | 0.08 | 0.05 | 1.05E-01 | 370 | 0.10 | 0.06 | 1.02E-01 | 210 | 0.07 | 0.03 | 1.48E-02 | 1214 |
| S_HDL_TG_pct | Relative lipoprotein lipid concentrations | CAC | 0.07 | 0.04 | 5.66E-02 | 643 | 0.04 | 0.05 | 4.63E-01 | 370 | 0.07 | 0.06 | 2.67E-01 | 210 | 0.07 | 0.03 | 1.51E-02 | 1214 |
| LA_pct | Fatty acids | AAC | -0.06 | 0.04 | 9.20E-02 | 643 | -0.10 | 0.06 | 7.72E-02 | 370 | NA | NA | NA | NA | -0.07 | 0.03 | 1.73E-02 | 1013 |
| M_VLDL_CE_pct | Relative lipoprotein lipid concentrations | CAC | -0.05 | 0.04 | 1.69E-01 | 643 | -0.05 | 0.05 | 2.76E-01 | 370 | -0.07 | 0.05 | 1.28E-01 | 210 | -0.06 | 0.02 | 1.82E-02 | 1214 |
| L_HDL_PL_pct | Relative lipoprotein lipid concentrations | CAC | 0.03 | 0.04 | 4.41E-01 | 643 | 0.10 | 0.05 | 3.69E-02 | 370 | 0.09 | 0.06 | 1.02E-01 | 210 | 0.07 | 0.03 | 1.85E-02 | 1214 |
| S_LDL_C_pct | Relative lipoprotein lipid concentrations | AAC | -0.05 | 0.04 | 1.44E-01 | 643 | -0.12 | 0.05 | 1.73E-02 | 370 | NA | NA | NA | NA | -0.08 | 0.03 | 1.87E-02 | 1013 |
| TG_by_PG | Other lipids | CAC | 0.08 | 0.04 | 4.18E-02 | 643 | 0.01 | 0.05 | 8.75E-01 | 370 | 0.09 | 0.06 | 1.47E-01 | 210 | 0.06 | 0.03 | 2.10E-02 | 1214 |
| M_VLDL_C_pct | Relative lipoprotein lipid concentrations | CAC | -0.05 | 0.04 | 2.02E-01 | 643 | -0.05 | 0.05 | 3.42E-01 | 370 | -0.10 | 0.06 | 8.61E-02 | 210 | -0.06 | 0.03 | 2.35E-02 | 1214 |
| L_HDL_L | Lipoprotein subclasses | CAC | -0.04 | 0.04 | 3.51E-01 | 643 | -0.07 | 0.05 | 1.40E-01 | 370 | -0.10 | 0.07 | 1.40E-01 | 210 | -0.06 | 0.03 | 2.56E-02 | 1214 |
| L_HDL_FC | Lipoprotein subclasses | CAC | -0.03 | 0.04 | 4.76E-01 | 643 | -0.09 | 0.05 | 5.70E-02 | 370 | -0.09 | 0.06 | 1.80E-01 | 210 | -0.06 | 0.03 | 2.71E-02 | 1214 |
| HDL_CE | Cholesteryl esters | CAC | -0.04 | 0.04 | 2.88E-01 | 643 | -0.06 | 0.05 | 2.31E-01 | 370 | -0.10 | 0.07 | 1.34E-01 | 210 | -0.06 | 0.03 | 2.74E-02 | 1214 |
| IDL_C_pct | Relative lipoprotein lipid concentrations | CAC | -0.04 | 0.04 | 2.28E-01 | 643 | -0.05 | 0.05 | 2.79E-01 | 370 | -0.07 | 0.06 | 1.92E-01 | 210 | -0.06 | 0.03 | 2.83E-02 | 1214 |
| Unsaturation | Fatty acids | CAC | -0.05 | 0.03 | 1.77E-01 | 643 | -0.04 | 0.05 | 4.69E-01 | 370 | -0.09 | 0.06 | 1.18E-01 | 210 | -0.06 | 0.03 | 2.91E-02 | 1214 |
| S_HDL_TG | Lipoprotein subclasses | CAC | 0.07 | 0.04 | 6.56E-02 | 643 | 0.03 | 0.05 | 5.84E-01 | 370 | 0.05 | 0.06 | 3.89E-01 | 210 | 0.06 | 0.03 | 3.03E-02 | 1214 |
| L_HDL_P | Lipoprotein subclasses | CAC | -0.03 | 0.04 | 4.35E-01 | 643 | -0.08 | 0.05 | 1.19E-01 | 370 | -0.10 | 0.07 | 1.46E-01 | 210 | -0.06 | 0.03 | 3.13E-02 | 1214 |
| S_LDL_C_pct | Relative lipoprotein lipid concentrations | CAC | -0.04 | 0.04 | 2.26E-01 | 643 | -0.04 | 0.05 | 3.60E-01 | 370 | -0.08 | 0.06 | 1.41E-01 | 210 | -0.06 | 0.03 | 3.15E-02 | 1214 |
| IDL_TG | Lipoprotein subclasses | AVC | 0.12 | 0.05 | 1.52E-02 | 643 | 0.04 | 0.05 | 3.58E-01 | 370 | NA | NA | NA | NA | 0.08 | 0.04 | 3.25E-02 | 1013 |
| Citrate | Glycolysis related metabolites | AAC | 0.05 | 0.04 | 1.33E-01 | 643 | 0.08 | 0.05 | 1.19E-01 | 370 | NA | NA | NA | NA | 0.06 | 0.03 | 3.29E-02 | 1013 |
| L_HDL_PL | Lipoprotein subclasses | CAC | -0.03 | 0.04 | 3.64E-01 | 643 | -0.07 | 0.05 | 1.79E-01 | 370 | -0.09 | 0.07 | 1.67E-01 | 210 | -0.06 | 0.03 | 3.75E-02 | 1214 |
| HDL_C | Cholesterol | CAC | -0.04 | 0.04 | 3.65E-01 | 643 | -0.06 | 0.05 | 2.09E-01 | 370 | -0.10 | 0.07 | 1.55E-01 | 210 | -0.06 | 0.03 | 3.81E-02 | 1214 |
| S_HDL_CE_pct | Relative lipoprotein lipid concentrations | AVC | -0.05 | 0.05 | 2.74E-01 | 643 | -0.08 | 0.04 | 7.17E-02 | 370 | NA | NA | NA | NA | -0.06 | 0.03 | 3.93E-02 | 1013 |
| XL_HDL_CE | Lipoprotein subclasses | CAC | -0.02 | 0.04 | 5.94E-01 | 643 | -0.11 | 0.05 | 2.50E-02 | 370 | -0.10 | 0.06 | 1.32E-01 | 210 | -0.07 | 0.03 | 4.25E-02 | 1214 |
| M_HDL_TG_pct | Relative lipoprotein lipid concentrations | CAC | 0.06 | 0.04 | 9.70E-02 | 643 | 0.03 | 0.05 | 4.79E-01 | 370 | 0.03 | 0.06 | 5.83E-01 | 210 | 0.05 | 0.03 | 4.33E-02 | 1214 |
| S_LDL_PL_pct | Relative lipoprotein lipid concentrations | AAC | 0.05 | 0.03 | 1.90E-01 | 643 | 0.10 | 0.06 | 8.22E-02 | 370 | NA | NA | NA | NA | 0.06 | 0.03 | 4.35E-02 | 1013 |
| L_LDL_C_pct | Relative lipoprotein lipid concentrations | AVC | -0.07 | 0.05 | 1.38E-01 | 643 | -0.06 | 0.04 | 1.70E-01 | 370 | NA | NA | NA | NA | -0.06 | 0.03 | 4.37E-02 | 1013 |
| VLDL_size | Lipoprotein particle sizes | CAC | 0.05 | 0.04 | 1.34E-01 | 643 | 0.00 | 0.05 | 9.47E-01 | 370 | 0.12 | 0.06 | 5.50E-02 | 210 | 0.05 | 0.03 | 4.52E-02 | 1214 |

Bold indicates significant association from multi-test correction (P-value < 4.39×10⁻⁴). NMR metabolic biomarkers: the biomarkers used as exposures. Model 2 was adjusted for age, sex, lipid-lowering medication, hypertension, diabetes, hypercholesterolemia, smoking, BMI, and history of cardiovascular disease.

Metabolic biomarkers: the biomarkers used as exposures.

Metabolic group: the NMR biomarker subgroups.

Calcification: site-specific calcification used as the outcome, CAC: coronary artery calcification; AAC: aortic arch calcification; AVC: aortic valve calcification.

Beta: estimated standard deviation changes of calcification across one standard deviation increase of metabolic biomarkers.

SE: standard error of the estimated effect size.

P-value: strength of evidence against the null hypothesis of no association between biomarker and calcification.

N: number of participants included in the analysis.

**Supplementary Table 5:** **Nominally significant association (P-value < 0.05) between NMR metabolic biomarkers and calcification in sex-stratified analysis (male only).**

| **Metabolic biomarkers** | **Metabolic group** | **outcome** |  |  | **Model 1** |  |  |  | **Model 2** |  |
| --- | --- | --- | --- | --- | --- | --- | --- | --- | --- | --- |
|  |  |  | **Beta** | **SE** | **P-value** | **N** | **Beta** | **SE** | **P-value** | **N** |
| GlycA | Inflammation | CAC | **0.12** | **0.03** | **6.04E-04** | **656** | **0.13** | **0.04** | **3.10E-04** | **601** |
| GlycA | Inflammation | AAC | **0.15** | **0.04** | **2.71E-04** | **518** | 0.13 | 0.05 | 3.81E-03 | 498 |
| LDL_size | Lipoprotein particle sizes | CAC | -0.09 | 0.03 | 5.72E-03 | 656 | -0.11 | 0.03 | 2.13E-03 | 601 |
| LA_pct | Fatty acids | AAC | -0.12 | 0.04 | 2.43E-03 | 518 | -0.11 | 0.05 | 1.83E-02 | 498 |
| LA_pct | Fatty acids | CAC | -0.10 | 0.03 | 3.04E-03 | 656 | -0.09 | 0.04 | 1.04E-02 | 601 |
| L_HDL_PL_pct | Relative lipoprotein lipid concentrations | CAC | 0.09 | 0.03 | 3.72E-03 | 656 | 0.08 | 0.03 | 2.03E-02 | 601 |
| L_HDL_CE_pct | Relative lipoprotein lipid concentrations | CAC | -0.08 | 0.03 | 3.74E-03 | 656 | -0.07 | 0.03 | 2.26E-02 | 601 |
| XXL_VLDL_FC_pct | Relative lipoprotein lipid concentrations | CAC | -0.12 | 0.04 | 4.19E-03 | 518 | -0.11 | 0.04 | 8.85E-03 | 498 |
| Omega_6_pct | Fatty acids | CAC | -0.09 | 0.03 | 5.09E-03 | 656 | -0.09 | 0.04 | 9.52E-03 | 601 |
| Glucose | Glycolysis related metabolites | CAC | 0.10 | 0.04 | 6.30E-03 | 656 | 0.10 | 0.04 | 1.61E-02 | 601 |
| S_LDL_CE_pct | Relative lipoprotein lipid concentrations | AAC | -0.10 | 0.04 | 1.65E-02 | 518 | -0.12 | 0.04 | 6.92E-03 | 498 |
| L_HDL_C_pct | Relative lipoprotein lipid concentrations | CAC | -0.07 | 0.03 | 8.36E-03 | 656 | -0.07 | 0.03 | 3.61E-02 | 601 |
| S_LDL_PL_pct | Relative lipoprotein lipid concentrations | AAC | 0.09 | 0.04 | 2.59E-02 | 518 | 0.12 | 0.05 | 9.81E-03 | 498 |
| Ala | Amino acids | CAC | 0.09 | 0.03 | 1.12E-02 | 656 | 0.08 | 0.04 | 3.45E-02 | 601 |
| PUFA_pct | Fatty acids | CAC | -0.08 | 0.03 | 2.04E-02 | 656 | -0.07 | 0.04 | 4.83E-02 | 601 |
| Acetate | Ketone bodies | AAC | -0.14 | 0.09 | 1.12E-01 | 518 | -0.10 | 0.05 | 2.52E-02 | 498 |
| Citrate | Glycolysis related metabolites | AAC | 0.07 | 0.04 | 7.14E-02 | 518 | 0.10 | 0.04 | 2.57E-02 | 498 |
| bOHbutyrate | Ketone bodies | AAC | 0.10 | 0.05 | 3.06E-02 | 518 | 0.10 | 0.05 | 4.66E-02 | 498 |
| M_HDL_PL | Lipoprotein subclasses | AVC | 0.10 | 0.05 | 4.58E-02 | 518 | 0.11 | 0.05 | 3.92E-02 | 498 |
| S_LDL_C_pct | Relative lipoprotein lipid concentrations | AAC | -0.12 | 0.06 | 3.95E-02 | 518 | -0.11 | 0.06 | 4.90E-02 | 498 |
| Glucose | Glycolysis related metabolites | AAC | 0.09 | 0.04 | 4.13E-02 | 518 | 0.03 | 0.09 | 6.96E-01 | 498 |
| Omega_6_pct | Fatty acids | AAC | -0.09 | 0.04 | 4.14E-02 | 518 | -0.06 | 0.05 | 2.01E-01 | 498 |
| S_HDL_PL | Lipoprotein subclasses | AVC | 0.10 | 0.05 | 4.20E-02 | 518 | 0.09 | 0.05 | 8.65E-02 | 498 |
| L_LDL_FC_pct | Relative lipoprotein lipid concentrations | CAC | -0.06 | 0.03 | 4.61E-02 | 656 | -0.06 | 0.03 | 7.20E-02 | 601 |
| PUFA_by_MUFA | Fatty acids | CAC | -0.07 | 0.03 | 4.68E-02 | 656 | -0.07 | 0.04 | 7.94E-02 | 601 |

Bold indicates significant association from multi-test correction (P-value < 4.39×10⁻⁴). Model 1 was adjusted for age and lipid-lowering medication. Model 2 was adjusted for age, lipid-lowering medication, hypertension, diabetes, hypercholesterolemia, smoking, BMI, and history of cardiovascular disease.

Metabolic biomarkers: the biomarkers used as exposures.

Metabolic group: the NMR biomarker subgroups.

Calcification: site-specific calcification used as the outcome, CAC: coronary artery calcification; AAC: aortic arch calcification; AVC: aortic valve calcification.

Beta: estimated standard deviation changes of calcification across one standard deviation increase of metabolic biomarkers.

SE: standard error of the estimated effect size.

P-value: strength of evidence against the null hypothesis of no association between biomarker and calcification.

N: number of participants included in the analysis.

**Supplementary Table 6:** **Nominally significant association (P-value < 0.05) between NMR metabolic biomarkers and calcification in sex-stratified analysis (female only).**

| **Metabolic biomarkers** | **Metabolic group** | **outcome** |  |  | **Model 1** |  |  |  | **Model 2** |  |
| --- | --- | --- | --- | --- | --- | --- | --- | --- | --- | --- |
|  |  |  | **Beta** | **SE** | **P-value** | **N** | **Beta** | **SE** | **P-value** | **N** |
| L_HDL_TG_pct | Relative lipoprotein lipid concentrations | CAC | 0.13 | 0.04 | 1.10E-03 | 663 | 0.13 | 0.04 | 1.56E-03 | 613 |
| L_LDL_C_pct | Relative lipoprotein lipid concentrations | CAC | -0.13 | 0.04 | 5.47E-04 | 663 | -0.12 | 0.04 | 2.66E-03 | 613 |
| XS_VLDL_CE_pct | Relative lipoprotein lipid concentrations | CAC | **-0.15** | **0.04** | **1.96E-04** | 663 | -0.13 | 0.04 | 2.70E-03 | 613 |
| XS_VLDL_C_pct | Relative lipoprotein lipid concentrations | CAC | **-0.15** | **0.04** | **2.12E-04** | 663 | -0.13 | 0.04 | 3.13E-03 | 613 |
| M_LDL_TG_pct | Relative lipoprotein lipid concentrations | CAC | 0.13 | 0.04 | 4.94E-04 | 663 | 0.10 | 0.04 | 9.58E-03 | 613 |
| L_LDL_TG_pct | Relative lipoprotein lipid concentrations | CAC | 0.13 | 0.04 | 5.23E-04 | 663 | 0.11 | 0.04 | 8.80E-03 | 613 |
| S_LDL_TG_pct | Relative lipoprotein lipid concentrations | CAC | 0.13 | 0.04 | 9.06E-04 | 663 | 0.11 | 0.04 | 6.40E-03 | 613 |
| IDL_TG_pct | Relative lipoprotein lipid concentrations | CAC | 0.13 | 0.04 | 1.06E-03 | 663 | 0.10 | 0.04 | 1.74E-02 | 613 |
| MUFA_pct | Fatty acids | CAC | 0.12 | 0.04 | 1.20E-03 | 663 | 0.11 | 0.04 | 4.65E-03 | 613 |
| PUFA_by_MUFA | Fatty acids | CAC | -0.12 | 0.04 | 1.26E-03 | 663 | -0.12 | 0.04 | 2.83E-03 | 613 |
| S_LDL_FC_pct | Relative lipoprotein lipid concentrations | CAC | -0.12 | 0.04 | 1.55E-03 | 663 | -0.11 | 0.04 | 4.03E-03 | 613 |
| IDL_FC_pct | Relative lipoprotein lipid concentrations | CAC | -0.12 | 0.04 | 1.74E-03 | 663 | -0.10 | 0.04 | 7.93E-03 | 613 |
| S_LDL_C_pct | Relative lipoprotein lipid concentrations | CAC | -0.11 | 0.03 | 1.94E-03 | 663 | -0.08 | 0.04 | 3.49E-02 | 613 |
| S_HDL_TG_pct | Relative lipoprotein lipid concentrations | CAC | 0.11 | 0.04 | 2.15E-03 | 663 | 0.11 | 0.04 | 5.60E-03 | 613 |
| S_VLDL_FC_pct | Relative lipoprotein lipid concentrations | CAC | -0.12 | 0.04 | 2.65E-03 | 663 | -0.10 | 0.04 | 1.50E-02 | 613 |
| M_HDL_CE | Lipoprotein subclasses | AVC | -0.09 | 0.05 | 8.34E-02 | 529 | -0.11 | 0.04 | 2.96E-03 | 515 |
| S_VLDL_PL_pct | Relative lipoprotein lipid concentrations | CAC | -0.11 | 0.04 | 3.09E-03 | 663 | -0.10 | 0.04 | 1.50E-02 | 613 |
| M_HDL_C | Lipoprotein subclasses | AVC | -0.09 | 0.06 | 9.91E-02 | 529 | -0.11 | 0.04 | 3.50E-03 | 515 |
| Glucose | Glycolysis related metabolites | CAC | 0.12 | 0.04 | 3.95E-03 | 663 | 0.11 | 0.04 | 1.14E-02 | 613 |
| PUFA_pct | Fatty acids | CAC | -0.11 | 0.04 | 4.05E-03 | 663 | -0.11 | 0.04 | 4.79E-03 | 613 |
| M_LDL_FC_pct | Relative lipoprotein lipid concentrations | CAC | -0.11 | 0.04 | 4.47E-03 | 663 | -0.10 | 0.04 | 1.42E-02 | 613 |
| L_VLDL_TG_pct | Relative lipoprotein lipid concentrations | AAC | -0.10 | 0.04 | 4.58E-03 | 529 | -0.10 | 0.04 | 5.38E-03 | 515 |
| XS_VLDL_TG_pct | Relative lipoprotein lipid concentrations | CAC | 0.11 | 0.04 | 4.74E-03 | 663 | 0.09 | 0.04 | 2.88E-02 | 613 |
| M_HDL_TG_pct | Relative lipoprotein lipid concentrations | CAC | 0.11 | 0.04 | 4.78E-03 | 663 | 0.10 | 0.04 | 9.16E-03 | 613 |
| DHA_pct | Fatty acids | CAC | -0.08 | 0.04 | 3.00E-02 | 663 | -0.11 | 0.04 | 4.97E-03 | 613 |
| M_HDL_C_pct | Relative lipoprotein lipid concentrations | CAC | -0.11 | 0.04 | 4.98E-03 | 663 | -0.11 | 0.04 | 8.56E-03 | 613 |
| M_HDL_P | Lipoprotein subclasses | AVC | -0.09 | 0.06 | 1.40E-01 | 529 | -0.11 | 0.04 | 5.05E-03 | 515 |
| XL_HDL_TG_pct | Relative lipoprotein lipid concentrations | CAC | 0.10 | 0.04 | 5.09E-03 | 663 | 0.10 | 0.04 | 8.82E-03 | 613 |
| M_HDL_CE_pct | Relative lipoprotein lipid concentrations | CAC | -0.11 | 0.04 | 5.26E-03 | 663 | -0.10 | 0.04 | 1.29E-02 | 613 |
| XXL_VLDL_FC | Lipoprotein subclasses | CAC | 0.10 | 0.04 | 6.33E-03 | 663 | 0.09 | 0.04 | 1.43E-02 | 613 |
| HDL_CE | Cholesteryl esters | AVC | -0.08 | 0.05 | 1.13E-01 | 529 | -0.10 | 0.04 | 6.41E-03 | 515 |
| XXL_VLDL_P | Lipoprotein subclasses | CAC | 0.09 | 0.03 | 7.08E-03 | 663 | 0.09 | 0.04 | 1.68E-02 | 613 |
| L_VLDL_FC_pct | Relative lipoprotein lipid concentrations | AAC | 0.10 | 0.04 | 7.10E-03 | 529 | 0.09 | 0.04 | 1.20E-02 | 515 |
| HDL_CE | Cholesteryl esters | CAC | -0.10 | 0.04 | 7.28E-03 | 663 | -0.11 | 0.04 | 8.01E-03 | 613 |
| S_HDL_CE_pct | Relative lipoprotein lipid concentrations | CAC | -0.10 | 0.04 | 7.59E-03 | 663 | -0.08 | 0.04 | 3.29E-02 | 613 |
| Unsaturation | Fatty acids | CAC | -0.09 | 0.04 | 1.40E-02 | 663 | -0.10 | 0.04 | 7.71E-03 | 613 |
| XXL_VLDL_L | Lipoprotein subclasses | CAC | 0.09 | 0.03 | 7.88E-03 | 663 | 0.08 | 0.04 | 2.25E-02 | 613 |
| M_HDL_CE | Lipoprotein subclasses | CAC | -0.10 | 0.04 | 8.13E-03 | 663 | -0.10 | 0.04 | 1.27E-02 | 613 |
| IDL_C_pct | Relative lipoprotein lipid concentrations | CAC | -0.10 | 0.04 | 8.16E-03 | 663 | -0.06 | 0.04 | 1.22E-01 | 613 |
| M_HDL_FC | Lipoprotein subclasses | AVC | -0.08 | 0.06 | 1.85E-01 | 529 | -0.10 | 0.04 | 8.45E-03 | 515 |
| HDL_P | Lipoprotein particle concentrations | AAC | -0.10 | 0.05 | 5.58E-02 | 529 | -0.12 | 0.05 | 8.48E-03 | 515 |
| Gly | Amino acids | CAC | -0.09 | 0.03 | 8.53E-03 | 663 | -0.09 | 0.03 | 1.01E-02 | 613 |
| HDL_C | Cholesterol | AVC | -0.08 | 0.05 | 1.52E-01 | 529 | -0.10 | 0.04 | 8.89E-03 | 515 |
| L_HDL_C | Lipoprotein subclasses | CAC | -0.10 | 0.04 | 1.48E-02 | 663 | -0.11 | 0.04 | 8.95E-03 | 613 |
| L_HDL_CE | Lipoprotein subclasses | CAC | -0.10 | 0.04 | 1.25E-02 | 663 | -0.11 | 0.04 | 9.51E-03 | 613 |
| M_HDL_PL | Lipoprotein subclasses | AVC | -0.08 | 0.06 | 1.30E-01 | 529 | -0.10 | 0.04 | 9.69E-03 | 515 |
| M_HDL_C | Lipoprotein subclasses | CAC | -0.10 | 0.04 | 9.74E-03 | 663 | -0.10 | 0.04 | 1.38E-02 | 613 |
| HDL_C | Cholesterol | CAC | -0.10 | 0.04 | 1.03E-02 | 663 | -0.10 | 0.04 | 9.75E-03 | 613 |
| M_HDL_PL | Lipoprotein subclasses | AAC | -0.10 | 0.05 | 2.77E-02 | 529 | -0.11 | 0.04 | 9.83E-03 | 515 |
| Omega_6_pct | Fatty acids | CAC | -0.10 | 0.04 | 9.92E-03 | 663 | -0.09 | 0.04 | 2.45E-02 | 613 |
| HDL_P | Lipoprotein particle concentrations | AVC | -0.07 | 0.07 | 3.08E-01 | 529 | -0.10 | 0.04 | 1.02E-02 | 515 |
| M_LDL_C_pct | Relative lipoprotein lipid concentrations | CAC | -0.08 | 0.03 | 1.07E-02 | 663 | -0.06 | 0.04 | 6.75E-02 | 613 |
| S_HDL_TG_pct | Relative lipoprotein lipid concentrations | AAC | 0.10 | 0.04 | 1.08E-02 | 529 | 0.09 | 0.04 | 3.13E-02 | 515 |
| M_HDL_L | Lipoprotein subclasses | AVC | -0.08 | 0.06 | 1.49E-01 | 529 | -0.10 | 0.04 | 1.11E-02 | 515 |
| M_HDL_L | Lipoprotein subclasses | AAC | -0.10 | 0.05 | 2.79E-02 | 529 | -0.11 | 0.04 | 1.12E-02 | 515 |
| Total_P | Lipoprotein particle concentrations | AAC | -0.10 | 0.06 | 9.51E-02 | 529 | -0.12 | 0.05 | 1.12E-02 | 515 |
| L_HDL_FC | Lipoprotein subclasses | CAC | -0.10 | 0.05 | 6.17E-02 | 663 | -0.11 | 0.04 | 1.16E-02 | 613 |
| XXL_VLDL_PL | Lipoprotein subclasses | CAC | 0.09 | 0.03 | 1.16E-02 | 663 | 0.08 | 0.04 | 2.38E-02 | 613 |
| M_HDL_CE | Lipoprotein subclasses | AAC | -0.10 | 0.04 | 1.55E-02 | 529 | -0.11 | 0.04 | 1.23E-02 | 515 |
| M_HDL_C | Lipoprotein subclasses | AAC | -0.10 | 0.04 | 1.97E-02 | 529 | -0.11 | 0.04 | 1.24E-02 | 515 |
| L_HDL_L | Lipoprotein subclasses | CAC | -0.09 | 0.04 | 2.39E-02 | 663 | -0.10 | 0.04 | 1.26E-02 | 613 |
| L_HDL_PL | Lipoprotein subclasses | CAC | -0.09 | 0.04 | 2.53E-02 | 663 | -0.10 | 0.04 | 1.28E-02 | 613 |
| L_VLDL_PL_pct | Relative lipoprotein lipid concentrations | AAC | 0.09 | 0.04 | 1.30E-02 | 529 | 0.08 | 0.04 | 3.23E-02 | 515 |
| M_HDL_P | Lipoprotein subclasses | AAC | -0.10 | 0.05 | 2.77E-02 | 529 | -0.11 | 0.04 | 1.34E-02 | 515 |
| S_HDL_C_pct | Relative lipoprotein lipid concentrations | CAC | -0.09 | 0.04 | 1.40E-02 | 663 | -0.09 | 0.04 | 2.22E-02 | 613 |
| DHA | Fatty acids | CAC | -0.06 | 0.05 | 1.52E-01 | 663 | -0.10 | 0.04 | 1.41E-02 | 613 |
| M_HDL_FC | Lipoprotein subclasses | AAC | -0.10 | 0.05 | 4.40E-02 | 529 | -0.11 | 0.05 | 1.45E-02 | 515 |
| L_HDL_TG_pct | Relative lipoprotein lipid concentrations | AVC | 0.09 | 0.04 | 1.48E-02 | 529 | 0.09 | 0.04 | 2.08E-02 | 515 |
| HDL_PL | Phospholipids | AVC | -0.08 | 0.06 | 1.91E-01 | 529 | -0.09 | 0.04 | 1.48E-02 | 515 |
| HDL_P | Lipoprotein particle concentrations | CAC | -0.09 | 0.04 | 1.51E-02 | 663 | -0.10 | 0.04 | 1.86E-02 | 613 |
| ApoB_by_ApoA1 | Apolipoproteins | AVC | 0.07 | 0.03 | 3.41E-02 | 529 | 0.08 | 0.03 | 1.51E-02 | 515 |
| HDL_L | Total lipids | AVC | -0.07 | 0.06 | 2.21E-01 | 529 | -0.09 | 0.04 | 1.53E-02 | 515 |
| S_HDL_CE | Lipoprotein subclasses | AAC | -0.08 | 0.04 | 5.51E-02 | 529 | -0.11 | 0.04 | 1.54E-02 | 515 |
| L_LDL_FC_pct | Relative lipoprotein lipid concentrations | CAC | -0.10 | 0.04 | 1.55E-02 | 663 | -0.08 | 0.04 | 7.61E-02 | 613 |
| TG_by_PG | Other lipids | CAC | 0.09 | 0.04 | 1.56E-02 | 663 | 0.09 | 0.04 | 2.63E-02 | 613 |
| S_HDL_C | Lipoprotein subclasses | AAC | -0.07 | 0.04 | 7.57E-02 | 529 | -0.11 | 0.04 | 1.58E-02 | 515 |
| XXL_VLDL_C | Lipoprotein subclasses | CAC | 0.08 | 0.03 | 1.63E-02 | 663 | 0.08 | 0.04 | 3.01E-02 | 613 |
| Total_P | Lipoprotein particle concentrations | CAC | -0.09 | 0.04 | 1.80E-02 | 663 | -0.10 | 0.04 | 1.69E-02 | 613 |
| ApoA1 | Apolipoproteins | AAC | -0.09 | 0.05 | 6.09E-02 | 529 | -0.10 | 0.04 | 1.69E-02 | 515 |
| L_HDL_P | Lipoprotein subclasses | CAC | -0.09 | 0.04 | 3.56E-02 | 663 | -0.10 | 0.04 | 1.72E-02 | 613 |
| M_HDL_C_pct | Relative lipoprotein lipid concentrations | AVC | -0.09 | 0.04 | 2.03E-02 | 529 | -0.09 | 0.04 | 1.77E-02 | 515 |
| L_HDL_CE_pct | Relative lipoprotein lipid concentrations | CAC | -0.13 | 0.05 | 1.78E-02 | 663 | -0.11 | 0.05 | 2.61E-02 | 613 |
| XS_VLDL_TG | Lipoprotein subclasses | AAC | 0.10 | 0.04 | 1.81E-02 | 529 | 0.07 | 0.04 | 9.16E-02 | 515 |
| M_HDL_CE_pct | Relative lipoprotein lipid concentrations | AVC | -0.09 | 0.04 | 1.81E-02 | 529 | -0.09 | 0.04 | 1.82E-02 | 515 |
| Total_P | Lipoprotein particle concentrations | AVC | -0.06 | 0.08 | 4.49E-01 | 529 | -0.10 | 0.04 | 1.89E-02 | 515 |
| Creatinine | Fluid balance | CAC | 0.14 | 0.06 | 1.96E-02 | 663 | 0.10 | 0.07 | 1.89E-01 | 613 |
| M_HDL_P | Lipoprotein subclasses | CAC | -0.09 | 0.04 | 1.99E-02 | 663 | -0.09 | 0.04 | 2.84E-02 | 613 |
| XXL_VLDL_PL_pct | Relative lipoprotein lipid concentrations | CAC | 0.08 | 0.04 | 2.72E-02 | 529 | 0.09 | 0.04 | 1.99E-02 | 515 |
| S_HDL_TG | Lipoprotein subclasses | CAC | 0.09 | 0.04 | 2.03E-02 | 663 | 0.08 | 0.04 | 3.46E-02 | 613 |
| S_HDL_TG_pct | Relative lipoprotein lipid concentrations | AVC | 0.08 | 0.04 | 2.17E-02 | 529 | 0.08 | 0.04 | 2.33E-02 | 515 |
| M_HDL_FC | Lipoprotein subclasses | CAC | -0.09 | 0.04 | 2.29E-02 | 663 | -0.10 | 0.04 | 2.19E-02 | 613 |
| HDL_FC | Free cholesterol | CAC | -0.08 | 0.04 | 4.25E-02 | 663 | -0.10 | 0.04 | 2.23E-02 | 613 |
| ApoA1 | Apolipoproteins | AVC | -0.07 | 0.06 | 2.64E-01 | 529 | -0.09 | 0.04 | 2.24E-02 | 515 |
| M_HDL_TG_pct | Relative lipoprotein lipid concentrations | AAC | 0.10 | 0.04 | 2.25E-02 | 529 | 0.08 | 0.04 | 6.16E-02 | 515 |
| XXL_VLDL_CE_pct | Relative lipoprotein lipid concentrations | CAC | -0.09 | 0.04 | 2.27E-02 | 529 | -0.08 | 0.04 | 2.59E-02 | 515 |
| Ile | Amino acids | CAC | 0.09 | 0.04 | 2.32E-02 | 663 | 0.08 | 0.04 | 7.52E-02 | 613 |
| HDL_PL | Phospholipids | AAC | -0.09 | 0.04 | 4.82E-02 | 529 | -0.10 | 0.04 | 2.32E-02 | 515 |
| S_HDL_CE | Lipoprotein subclasses | CAC | -0.08 | 0.04 | 2.32E-02 | 663 | -0.07 | 0.04 | 5.63E-02 | 613 |
| M_HDL_L | Lipoprotein subclasses | CAC | -0.09 | 0.04 | 2.36E-02 | 663 | -0.09 | 0.04 | 3.14E-02 | 613 |
| HDL_L | Total lipids | CAC | -0.09 | 0.04 | 2.69E-02 | 663 | -0.09 | 0.04 | 2.42E-02 | 613 |
| IDL_TG | Lipoprotein subclasses | AAC | 0.09 | 0.04 | 2.46E-02 | 529 | 0.06 | 0.05 | 1.60E-01 | 515 |
| S_HDL_P | Lipoprotein subclasses | AAC | -0.06 | 0.04 | 1.21E-01 | 529 | -0.10 | 0.04 | 2.46E-02 | 515 |
| L_HDL_CE | Lipoprotein subclasses | AVC | -0.08 | 0.04 | 3.23E-02 | 529 | -0.09 | 0.04 | 2.52E-02 | 515 |
| LA_pct | Fatty acids | CAC | -0.09 | 0.04 | 2.54E-02 | 663 | -0.10 | 0.05 | 5.32E-02 | 613 |
| HDL_L | Total lipids | AAC | -0.09 | 0.05 | 5.56E-02 | 529 | -0.10 | 0.04 | 2.54E-02 | 515 |
| HDL_CE | Cholesteryl esters | AAC | -0.09 | 0.04 | 3.37E-02 | 529 | -0.10 | 0.04 | 2.56E-02 | 515 |
| XS_VLDL_PL | Lipoprotein subclasses | AAC | 0.09 | 0.04 | 2.58E-02 | 529 | 0.08 | 0.04 | 7.60E-02 | 515 |
| M_HDL_TG_pct | Relative lipoprotein lipid concentrations | AVC | 0.08 | 0.04 | 2.69E-02 | 529 | 0.08 | 0.04 | 2.93E-02 | 515 |
| HDL_TG | Triglycerides | CAC | 0.09 | 0.04 | 2.71E-02 | 663 | 0.08 | 0.04 | 6.53E-02 | 613 |
| ApoA1 | Apolipoproteins | CAC | -0.08 | 0.04 | 2.81E-02 | 663 | -0.09 | 0.04 | 3.10E-02 | 613 |
| L_HDL_TG_pct | Relative lipoprotein lipid concentrations | AAC | 0.10 | 0.04 | 2.85E-02 | 529 | 0.08 | 0.05 | 9.00E-02 | 515 |
| HDL_C | Cholesterol | AAC | -0.09 | 0.04 | 4.55E-02 | 529 | -0.10 | 0.04 | 2.86E-02 | 515 |
| L_HDL_PL | Lipoprotein subclasses | AVC | -0.08 | 0.04 | 3.88E-02 | 529 | -0.09 | 0.04 | 2.87E-02 | 515 |
| XS_VLDL_PL_pct | Relative lipoprotein lipid concentrations | CAC | 0.09 | 0.05 | 4.85E-02 | 663 | 0.09 | 0.04 | 2.91E-02 | 613 |
| XXL_VLDL_CE | Lipoprotein subclasses | CAC | 0.07 | 0.03 | 2.95E-02 | 663 | 0.07 | 0.04 | 4.80E-02 | 613 |
| M_HDL_PL | Lipoprotein subclasses | CAC | -0.08 | 0.04 | 3.05E-02 | 663 | -0.08 | 0.04 | 4.08E-02 | 613 |
| S_HDL_L | Lipoprotein subclasses | AAC | -0.07 | 0.05 | 1.41E-01 | 529 | -0.10 | 0.05 | 3.21E-02 | 515 |
| HDL_PL | Phospholipids | CAC | -0.08 | 0.04 | 3.40E-02 | 663 | -0.09 | 0.04 | 3.21E-02 | 613 |
| HDL_FC | Free cholesterol | AVC | -0.06 | 0.06 | 3.45E-01 | 529 | -0.08 | 0.04 | 3.31E-02 | 515 |
| L_HDL_C | Lipoprotein subclasses | AVC | -0.08 | 0.04 | 4.28E-02 | 529 | -0.09 | 0.04 | 3.31E-02 | 515 |
| L_HDL_L | Lipoprotein subclasses | AVC | -0.07 | 0.04 | 5.28E-02 | 529 | -0.08 | 0.04 | 3.49E-02 | 515 |
| S_HDL_FC | Lipoprotein subclasses | AAC | -0.06 | 0.05 | 2.93E-01 | 529 | -0.10 | 0.05 | 3.61E-02 | 515 |
| S_VLDL_CE | Lipoprotein subclasses | AAC | 0.08 | 0.04 | 3.62E-02 | 529 | 0.07 | 0.04 | 9.28E-02 | 515 |
| L_LDL_C_pct | Relative lipoprotein lipid concentrations | AAC | -0.08 | 0.04 | 3.67E-02 | 529 | -0.09 | 0.04 | 4.17E-02 | 515 |
| L_LDL_TG | Lipoprotein subclasses | AAC | 0.09 | 0.04 | 3.68E-02 | 529 | 0.05 | 0.05 | 2.80E-01 | 515 |
| LDL_TG | Triglycerides | AAC | 0.09 | 0.04 | 3.77E-02 | 529 | 0.05 | 0.05 | 2.71E-01 | 515 |
| Citrate | Glycolysis related metabolites | CAC | 0.12 | 0.06 | 3.79E-02 | 663 | 0.08 | 0.05 | 9.79E-02 | 613 |
| ApoB_by_ApoA1 | Apolipoproteins | AAC | 0.08 | 0.04 | 3.87E-02 | 529 | 0.07 | 0.04 | 6.84E-02 | 515 |
| L_HDL_P | Lipoprotein subclasses | AVC | -0.07 | 0.04 | 7.71E-02 | 529 | -0.08 | 0.04 | 3.96E-02 | 515 |
| S_HDL_C | Lipoprotein subclasses | CAC | -0.07 | 0.04 | 3.97E-02 | 663 | -0.07 | 0.04 | 6.54E-02 | 613 |
| S_HDL_PL | Lipoprotein subclasses | AAC | -0.08 | 0.05 | 1.36E-01 | 529 | -0.11 | 0.05 | 4.16E-02 | 515 |
| M_VLDL_CE_pct | Relative lipoprotein lipid concentrations | CAC | -0.10 | 0.05 | 4.16E-02 | 663 | -0.08 | 0.04 | 5.67E-02 | 613 |
| Total_TG | Triglycerides | CAC | 0.08 | 0.04 | 4.20E-02 | 663 | 0.07 | 0.04 | 8.39E-02 | 613 |
| M_HDL_FC_pct | Relative lipoprotein lipid concentrations | CAC | -0.08 | 0.07 | 2.37E-01 | 663 | -0.10 | 0.05 | 4.25E-02 | 613 |
| XS_VLDL_FC | Lipoprotein subclasses | AAC | 0.08 | 0.04 | 4.27E-02 | 529 | 0.07 | 0.04 | 1.03E-01 | 515 |
| S_HDL_CE | Lipoprotein subclasses | AVC | -0.05 | 0.04 | 1.43E-01 | 529 | -0.07 | 0.04 | 4.36E-02 | 515 |
| M_HDL_PL_pct | Relative lipoprotein lipid concentrations | CAC | 0.08 | 0.04 | 4.39E-02 | 663 | 0.10 | 0.06 | 1.03E-01 | 613 |
| XS_VLDL_L | Lipoprotein subclasses | AAC | 0.08 | 0.04 | 4.50E-02 | 529 | 0.07 | 0.04 | 1.26E-01 | 515 |
| XL_HDL_FC_pct | Relative lipoprotein lipid concentrations | CAC | 0.08 | 0.05 | 1.17E-01 | 663 | 0.11 | 0.06 | 4.52E-02 | 613 |
| S_VLDL_TG | Lipoprotein subclasses | CAC | 0.07 | 0.04 | 4.57E-02 | 663 | 0.06 | 0.04 | 1.44E-01 | 613 |
| M_LDL_TG | Lipoprotein subclasses | AAC | 0.09 | 0.04 | 4.63E-02 | 529 | 0.05 | 0.05 | 2.96E-01 | 515 |
| XXL_VLDL_C_pct | Relative lipoprotein lipid concentrations | CAC | -0.08 | 0.04 | 4.79E-02 | 529 | -0.07 | 0.04 | 5.50E-02 | 515 |
| XS_VLDL_FC | Lipoprotein subclasses | AVC | 0.06 | 0.06 | 3.02E-01 | 529 | 0.07 | 0.04 | 4.81E-02 | 515 |
| M_VLDL_C_pct | Relative lipoprotein lipid concentrations | CAC | -0.08 | 0.04 | 4.85E-02 | 663 | -0.07 | 0.04 | 8.34E-02 | 613 |
| IDL_C_pct | Relative lipoprotein lipid concentrations | AAC | -0.08 | 0.04 | 4.86E-02 | 529 | -0.06 | 0.04 | 1.48E-01 | 515 |
| HDL_FC | Free cholesterol | AAC | -0.08 | 0.05 | 1.17E-01 | 529 | -0.09 | 0.05 | 4.97E-02 | 515 |
| IDL_CE_pct | Relative lipoprotein lipid concentrations | AAC | -0.08 | 0.04 | 4.99E-02 | 529 | -0.07 | 0.04 | 1.06E-01 | 515 |

Bold indicates significant association from multi-test correction (P-value < 4.39×10⁻⁴). Model 1 was adjusted for age and lipid-lowering medication. Model 2 was adjusted for age, lipid-lowering medication, hypertension, diabetes, hypercholesterolemia, smoking, BMI, and history of cardiovascular disease.

Metabolic biomarkers: the biomarkers used as exposures.

Metabolic group: the NMR biomarker subgroups.

Calcification: site-specific calcification used as the outcome, CAC: coronary artery calcification; AAC: aortic arch calcification; AVC: aortic valve calcification.

Beta: estimated standard deviation changes of calcification across one standard deviation increase of metabolic biomarkers.

SE: standard error of the estimated effect size.

P-value: strength of evidence against the null hypothesis of no association between biomarker and calcification.

N: number of participants included in the analysis.

**Supplementary Table 7: Metabolic biomarkers significantly associated with calcification from sensitivity analysis by excluding participants with the history of cardiovascular diseases.**

| **Metabolic biomarkers** | **Beta** | **SE** | **P-value** | **N** | **Outcomes** | **Groups** |
| --- | --- | --- | --- | --- | --- | --- |
| PUFA_pct | -0.10 | 0.03 | 3.50E-04 | 1109 | CAC | Overall |
| PUFA_by_MUFA | -0.10 | 0.03 | 2.40E-04 | 1109 | CAC | Overall |
| S_LDL_C_pct | -0.12 | 0.03 | 2.82E-04 | 914 | AAC | Overall |

Significant association threshold from multi-test correction (P-value < 4.39×10⁻⁴). Regression analyses were adjusted for age, sex (overall analysis), lipid-lowering medication use, hypertension, diabetes, hypercholesterolemia, smoking status, and BMI.

Metabolic biomarkers: the biomarkers used as exposures.

Calcification: site-specific calcification used as the outcome, CAC: coronary artery calcification; AAC: aortic arch calcification; AVC: aortic valve calcification.

Beta: estimated standard deviation changes of calcification across one standard deviation increase of metabolic biomarkers.

SE: standard error of the estimated effect size.

P-value: strength of evidence against the null hypothesis of no association between biomarker and calcification.

N: number of participants included in the analysis.

**Supplementary Table 8:** **Post-hoc sensitivity analysis of detectable standardized effect sizes for calcification at coronary artery (CAC), aortic arch (AAC), and aortic valve (AVC).**

|  | CAC | AAC/AVC |
| --- | --- | --- |
| All | 0.121 | 0.136 |
| Men | 0.174 | 0.196 |
| Women | 0.173 | 0.194 |

The post-hoc sensitivity analyses were performed assuming 80% statistical power, a multiple-testing–adjusted significance threshold of 4.39×10⁻⁴, one predictor per outcome, and the available participants as the effective sample size.

**Supplementary Table 9: The variants included as genetic instruments in two-sample Mendelian randomization.**

| **Metabolic group** | **Metabolic biomarkers** | **IVs** |
| --- | --- | --- |
| Fatty acids | Omega_6_pct | rs10733306, rs10773049, rs111351217, rs11508026, rs1168032, rs116843064, rs11976955, rs11998606, rs12419462, rs1260326, rs1316753, rs13389219, rs139974673, rs1471251, rs1546224, rs174528, rs1801689, rs182050989, rs2068888, rs261290, rs28601761, rs295268, rs2972140, rs328, rs3812316, rs4665710, rs499765, rs56001710, rs58489806, rs6073958, rs6658257, rs684773, rs6905288, rs6938550, rs72836561, rs73412716, rs7402939, rs78058190, rs964184 |
| Fatty acids | PUFA_by_MUFA | rs10937924, rs11207994, rs11235, rs11644601, rs116843064, rs1260326, rs13389219, rs1471251, rs1495741, rs150844304, rs1801689, rs182549, rs2035816, rs2049245, rs2081194, rs2229738, rs28601761, rs2925979, rs2972140, rs35163364, rs3789849, rs3812316, rs56001710, rs56156922, rs603424, rs6073958, rs61781371, rs632057, rs673548, rs67981690, rs6866758, rs72836561, rs7402939, rs78058190, rs79293855, rs79598313, rs9419746, rs964184, rs998584 |
| Fatty acids | PUFA_pct | rs10020067, rs10746732, rs11644601, rs1168041, rs116843064, rs1180384, rs12419462, rs1260326, rs13389219, rs139974673, rs1495741, rs150543195, rs174564, rs182549, rs2035816, rs2081194, rs2229738, rs261290, rs28601761, rs2972140, rs3812316, rs56001710, rs56156922, rs6073958, rs632057, rs655720, rs6606731, rs673548, rs6905288, rs72836561, rs7402939, rs78058190, rs79598313, rs9419746, rs964184 |
| Relative lipoprotein lipid concentrations | L_HDL_CE_pct | rs10750766, rs10773049, rs10774625, rs10937924, rs11077783, rs11207994, rs116843064, rs11922042, rs12446515, rs1260326, rs13107325, rs1316753, rs13389219, rs139953093, rs150844304, rs1645787, rs174584, rs1982151, rs2035403, rs2068888, rs2175055, rs2414577, rs2745353, rs28601761, rs28752510, rs2925979, rs2972140, rs328, rs3741414, rs3812316, rs4519692, rs4656292, rs4800392, rs56001710, rs58542926, rs62397245, rs628418, rs6658257, rs668459, rs67916282, rs6796333, rs6825776, rs6864091, rs7012637, rs7132434, rs72836348, rs72836561, rs7357754, rs76070947, rs76381434, rs78058190, rs7810507, rs79598313, rs8044652, rs8126001, rs964184, rs998584 |
| Relative lipoprotein lipid concentrations | L_HDL_C_pct | rs1057208, rs10750766, rs10773049, rs10798586, rs10937924, rs11207994, rs116843064, rs11922042, rs1260326, rs13107325, rs13118477, rs13389219, rs146077850, rs150844304, rs151014368, rs1645787, rs17052058, rs174554, rs1982151, rs2035403, rs2068888, rs2229357, rs236651, rs28601761, rs28752510, rs289703, rs2925979, rs2943654, rs35246381, rs3767962, rs3812316, rs4519692, rs4656292, rs4800392, rs58542926, rs653178, rs668459, rs675849, rs67916282, rs680379, rs6864091, rs7012637, rs7132434, rs72786786, rs72836348, rs72836561, rs72959041, rs7357754, rs76070947, rs77271869, rs78058190, rs7810507, rs79598313, rs8126001, rs953618, rs964184, rs9844972, rs998584 |
| Relative lipoprotein lipid concentrations | M_LDL_FC_pct | rs10750766, rs10822168, rs11591147, rs11644601, rs116843064, rs1260326, rs13108218, rs1316753, rs13389219, rs1471251, rs150844304, rs174554, rs1801689, rs2035816, rs2068888, rs2275647, rs2292643, rs261290, rs2678379, rs28601761, rs2925979, rs2972140, rs328, rs34417180, rs35163364, rs37538, rs3789849, rs3812316, rs41284486, rs4704834, rs56205943, rs56287412, rs59476633, rs632057, rs67408364, rs6796333, rs684773, rs6971365, rs7133378, rs7157785, rs72836561, rs72959041, rs7402939, rs7679, rs78058190, rs79598313, rs821840, rs964184, rs998584 |
| Relative lipoprotein lipid concentrations | M_HDL_CE_pct | rs1022186, rs1128249, rs116843064, rs12601919, rs1260326, rs13107325, rs146203232, rs1471251, rs150844304, rs150911013, rs16961845, rs174544, rs1800961, rs2068888, rs2267373, rs2292643, rs261290, rs28601761, rs28712486, rs28752510, rs2925979, rs2943650, rs328, rs34931250, rs35292873, rs431001, rs4660303, rs4665710, rs55747707, rs56001710, rs56205943, rs61838776, rs632057, rs6796333, rs6942635, rs6971365, rs7012637, rs7133378, rs7134375, rs72836561, rs77244967, rs78058190, rs79598313, rs8107974, rs821840, rs830083, rs9556403, rs964184, rs9647335, rs998584 |
| Lipoprotein subclasses | L_HDL_CE | rs1024137, rs10468017, rs11045171, rs11075253, rs11122450, rs112875651, rs116843064, rs1177562, rs11922042, rs1215112, rs12510382, rs1260326, rs13107325, rs13118477, rs13389219, rs145276599, rs145391587, rs150429317, rs174576, rs17585887, rs190543502, rs2159502, rs2229357, rs2307111, rs2642438, rs2737205, rs2740488, rs289703, rs2925339, rs2925979, rs3110291, rs333947, rs34062580, rs36226283, rs3922628, rs4239651, rs4418728, rs4922787, rs4986970, rs5082, rs5167, rs57912727, rs59104589, rs6073958, rs61781392, rs61854123, rs653178, rs6694102, rs673548, rs688671, rs6993128, rs7012814, rs705379, rs72786786, rs72823014, rs72836561, rs737337, rs759819, rs77960347, rs78058190, rs7810507, rs79598313, rs80236739, rs8064100, rs849336, rs921919, rs9491697, rs964184, rs9769088, rs9844972, rs998584 |
| Relative lipoprotein lipid concentrations | M_HDL_C_pct | rs1022186, rs10750766, rs1128249, rs116843064, rs1215112, rs12601919, rs1260326, rs13107325, rs13284054, rs150844304, rs16961845, rs174580, rs1761457, rs1802539, rs2068888, rs2112347, rs2267375, rs2281721, rs28601761, rs289703, rs2925979, rs2943650, rs328, rs34931250, rs35853021, rs3775228, rs4660303, rs4800392, rs4969179, rs55747707, rs56205943, rs6073958, rs61838776, rs632057, rs673548, rs6796333, rs6942635, rs6971365, rs7012637, rs7133378, rs7134375, rs72786786, rs72823020, rs72836561, rs78058190, rs7924036, rs79598313, rs80237246, rs8044652, rs830083, rs9556403, rs964184, rs9647335, rs998584 |
| Inflammation | GlycA | rs112875651, rs1132812, rs113354603, rs116843064, rs12032372, rs12208357, rs12239736, rs1260326, rs13108218, rs149807892, rs150844304, rs17105232, rs1801020, rs1801689, rs182050989, rs2070634, rs2294915, rs2445818, rs28929474, rs2925979, rs55780214, rs56188865, rs58542926, rs59296513, rs59774409, rs62466318, rs6452937, rs6601299, rs6734238, rs676210, rs687621, rs72801474, rs7697204, rs79287178, rs964184 |
| Lipoprotein subclasses | L_HDL_C | rs1024137, rs10468017, rs1057208, rs11045171, rs11122450, rs112875651, rs116843064, rs1177562, rs11922042, rs1215112, rs12510382, rs1260326, rs13107325, rs13118477, rs13389219, rs145276599, rs145391587, rs174576, rs17585887, rs190543502, rs2159502, rs2229357, rs2307111, rs2642438, rs2737205, rs2740488, rs289703, rs2925339, rs2925979, rs333947, rs34062580, rs36226283, rs3922628, rs4418728, rs4922787, rs4986970, rs5082, rs57912727, rs59104589, rs61781392, rs61854123, rs6498540, rs653178, rs6694102, rs673548, rs688671, rs6993128, rs7012814, rs705379, rs72786786, rs72823014, rs72836561, rs737337, rs759819, rs76070947, rs77960347, rs78058190, rs7810507, rs79598313, rs80236739, rs8064100, rs849336, rs921919, rs9491697, rs964184, rs9769088, rs9844972, rs998584 |
| Relative lipoprotein lipid concentrations | L_HDL_TG_pct | rs10882140, rs11122450, rs1128249, rs113414093, rs11644601, rs1168015, rs116843064, rs12601919, rs1260326, rs13108218, rs13118477, rs1471251, rs150844304, rs174580, rs2186037, rs2267373, rs261290, rs28383314, rs28601761, rs289703, rs2925979, rs2943650, rs328, rs34060476, rs35518360, rs37538, rs3996352, rs4569100, rs4665710, rs4969144, rs56001710, rs56205943, rs58542926, rs6073958, rs61232586, rs61838776, rs668459, rs6694102, rs6796333, rs7012637, rs7133378, rs7134375, rs72786786, rs72823020, rs72836561, rs76381434, rs79598313, rs80237246, rs8044652, rs8126001, rs964184, rs998584 |
| Lipoprotein subclasses | L_HDL_FC | rs1024137, rs10457487, rs1057208, rs10899133, rs11045171, rs112875651, rs116843064, rs11751347, rs11922042, rs1215112, rs12510382, rs1260326, rs13107325, rs13389219, rs141949189, rs145276599, rs145391587, rs1457489, rs1546954, rs190543502, rs193084249, rs1973688, rs2307111, rs2642438, rs2737205, rs2740488, rs289703, rs2925339, rs2925979, rs333947, rs34060476, rs36226283, rs3735687, rs3809113, rs4418728, rs4986970, rs5082, rs56959712, rs57912727, rs59104589, rs61781392, rs6498540, rs6694102, rs676210, rs7012814, rs705379, rs7241918, rs72786786, rs72823014, rs72836561, rs7308864, rs737337, rs76070947, rs77960347, rs78058190, rs7810507, rs79744701, rs80236739, rs8064100, rs849336, rs921919, rs964184, rs998584 |
| Other lipids | TG_by_PG | rs1022186, rs10274367, rs10750766, rs1077834, rs10838681, rs10882140, rs11122450, rs1128249, rs11609805, rs11644601, rs116843064, rs11976955, rs11998606, rs1260326, rs13107325, rs13108218, rs1471251, rs150844304, rs1601935, rs174580, rs1801689, rs2131925, rs2186037, rs2267375, rs2642438, rs2803619, rs28601761, rs2925979, rs2943650, rs328, rs35223533, rs35678857, rs37538, rs3764261, rs3812316, rs4698315, rs4704834, rs4871844, rs4969179, rs56001710, rs6073958, rs61781371, rs61838776, rs632057, rs6542680, rs676210, rs6796333, rs684773, rs6942635, rs7133378, rs7134375, rs7140110, rs72836561, rs78058190, rs78795332, rs79598313, rs964184, rs9844972, rs9889584, rs998584 |
| Lipoprotein subclasses | S_HDL_TG | rs10750766, rs10761716, rs10838681, rs113414093, rs11644601, rs116843064, rs117209788, rs11976955, rs12212507, rs12313762, rs12601919, rs1260326, rs13108218, rs1316753, rs13389219, rs1471251, rs149480839, rs150844304, rs174580, rs2068888, rs2070971, rs261290, rs28383314, rs28601761, rs2925979, rs2972140, rs328, rs34682685, rs3812316, rs4665710, rs4704834, rs56001710, rs632057, rs6796333, rs72836561, rs7679, rs76895963, rs7837587, rs79598313, rs8107974, rs8126001, rs821840, rs863750, rs964184, rs9749209, rs9844972, rs9889584, rs998584 |
| Lipoprotein particle sizes | VLDL_size | rs10401038, rs10468017, rs10750766, rs10773049, rs10786069, rs11075253, rs11122450, rs116843064, rs117209788, rs11903847, rs11976955, rs1260326, rs13108218, rs13115795, rs13389219, rs150844304, rs174553, rs17585887, rs1801689, rs1867172, rs2292644, rs2702552, rs2745353, rs28601761, rs2925979, rs2960834, rs2972140, rs2980755, rs328, rs34682685, rs35678857, rs3812316, rs3963364, rs4704834, rs4775090, rs5023763, rs5112, rs56001710, rs59296513, rs61587941, rs6689335, rs676210, rs7205804, rs72836561, rs7679, rs76873953, rs78058190, rs7924036, rs79598313, rs849327, rs964184, rs9968117, rs998584 |
| Lipoprotein subclasses | XL_HDL_C | rs10468017, rs10750766, rs10773049, rs10935473, rs10937924, rs11048457, rs11075253, rs11591147, rs116843064, rs1177562, rs11922042, rs1215112, rs1260326, rs13107325, rs13118477, rs13389219, rs17585887, rs190543502, rs2068888, rs2242332, rs2281719, rs2642438, rs2740488, rs289703, rs2909211, rs2925979, rs2943654, rs2954029, rs34062580, rs3809113, rs4699716, rs4841605, rs5082, rs577195, rs57912727, rs59104589, rs6032614, rs6065904, rs653178, rs6694102, rs6864091, rs705379, rs72786786, rs72836561, rs72959041, rs77960347, rs78058190, rs80236739, rs849336, rs921919, rs964184, rs9769088, rs9844972, rs998584 |
| Relative lipoprotein lipid concentrations | M_HDL_TG_pct | rs1022186, rs10882140, rs112001035, rs11228377, rs1128249, rs116843064, rs12446515, rs12601919, rs1260326, rs12747505, rs13107325, rs143843429, rs1471251, rs150844304, rs150911013, rs16961845, rs174580, rs2267375, rs261290, rs2800708, rs28601761, rs2925979, rs2943650, rs328, rs37538, rs3812316, rs4660303, rs4665710, rs4969179, rs56001710, rs56205943, rs6073958, rs632057, rs6796333, rs6942635, rs6971365, rs7012637, rs7133378, rs7134375, rs72823020, rs72836561, rs78058190, rs79598313, rs80237246, rs8044652, rs8107974, rs830083, rs964184, rs9647335, rs998584 |

Metabolic biomarkers: the biomarkers used as exposures.

Metabolic group: the NMR biomarker subgroups.

IVs: the kept instruments from harmonization and outlier test.

**Supplementary Table 10. Two-sample Mendelian randomization (MR) indicates the causal associations between NMR 17 metabolic biomarkers and coronary artery calcification**

| **Metabolic group** | **Metabolic biomarkers** | **F-Statistics** | **Method** | **IVs** | **Beta** | **SE** | **P-value** |
| --- | --- | --- | --- | --- | --- | --- | --- |
| Fatty acids | Omega_6_pct | 108.79 | MR Egger | 37 | -0.31 | 0.14 | 3.01E-02 |
|  |  |  | Inverse variance weighted | 37 | -0.35 | 0.08 | 1.05E-05 |
|  |  |  | Weighted median | 37 | -0.35 | 0.11 | 2.13E-03 |
| Fatty acids | PUFA_by_MUFA | 100.75 | Weighted median | 36 | -0.36 | 0.12 | 2.96E-03 |
|  |  |  | Inverse variance weighted | 36 | -0.36 | 0.08 | 1.77E-05 |
|  |  |  | MR Egger | 36 | -0.30 | 0.15 | 5.72E-02 |
| Fatty acids | PUFA_pct | 103.19 | Weighted median | 33 | -0.32 | 0.14 | 2.46E-02 |
|  |  |  | Inverse variance weighted | 33 | -0.35 | 0.09 | 7.97E-05 |
|  |  |  | MR Egger | 33 | -0.08 | 0.16 | 6.19E-01 |
| Relative lipoprotein lipid concentrations | L_HDL_CE_pct | 128.65 | Inverse variance weighted | 55 | -0.25 | 0.06 | 8.96E-05 |
|  |  |  | Weighted median | 55 | -0.25 | 0.08 | 3.54E-03 |
|  |  |  | MR Egger | 55 | -0.24 | 0.10 | 2.02E-02 |
| Relative lipoprotein lipid concentrations | L_HDL_C_pct | 123.53 | Weighted median | 57 | -0.24 | 0.09 | 6.45E-03 |
|  |  |  | Inverse variance weighted | 57 | -0.27 | 0.06 | 9.72E-06 |
|  |  |  | MR Egger | 57 | -0.24 | 0.10 | 1.57E-02 |
| Relative lipoprotein lipid concentrations | M_LDL_FC_pct | 143.73 | Weighted median | 47 | -0.28 | 0.09 | 1.76E-03 |
|  |  |  | MR Egger | 47 | -0.24 | 0.11 | 2.74E-02 |
|  |  |  | Inverse variance weighted | 47 | -0.24 | 0.07 | 2.69E-04 |
| Relative lipoprotein lipid concentrations | M_HDL_CE_pct | 152.54 | Inverse variance weighted | 48 | -0.28 | 0.06 | 2.83E-06 |
|  |  |  | Weighted median | 48 | -0.24 | 0.09 | 5.49E-03 |
|  |  |  | MR Egger | 48 | -0.25 | 0.10 | 1.30E-02 |
| Relative lipoprotein lipid concentrations | M_HDL_C_pct | 147.12 | Inverse variance weighted | 53 | -0.24 | 0.07 | 2.14E-04 |
|  |  |  | Weighted median | 53 | -0.24 | 0.09 | 5.87E-03 |
|  |  |  | MR Egger | 53 | -0.25 | 0.11 | 2.22E-02 |
| Lipoprotein subclasses | L_HDL_CE | 129.13 | Inverse variance weighted | 71 | -0.21 | 0.06 | 4.02E-04 |
|  |  |  | MR Egger | 71 | -0.17 | 0.09 | 7.30E-02 |
|  |  |  | Weighted median | 71 | -0.18 | 0.09 | 4.20E-02 |
| Inflammation | GlycA | 100.41 | MR Egger | 35 | 0.61 | 0.17 | 1.16E-03 |
|  |  |  | Weighted median | 35 | 0.38 | 0.12 | 1.75E-03 |
|  |  |  | Inverse variance weighted | 35 | 0.36 | 0.10 | 2.11E-04 |
| Lipoprotein subclasses | L_HDL_C | 132.10 | MR Egger | 68 | -0.16 | 0.09 | 9.56E-02 |
|  |  |  | Weighted median | 68 | -0.17 | 0.09 | 4.32E-02 |
|  |  |  | Inverse variance weighted | 68 | -0.21 | 0.06 | 4.93E-04 |
| Relative lipoprotein lipid concentrations | L_HDL_TG_pct | 173.23 | Weighted median | 50 | 0.24 | 0.08 | 2.75E-03 |
|  |  |  | Inverse variance weighted | 50 | 0.26 | 0.06 | 4.92E-06 |
|  |  |  | MR Egger | 50 | 0.16 | 0.09 | 8.20E-02 |
| Other lipids | TG_by_PG | 126.73 | MR Egger | 58 | 0.30 | 0.10 | 4.01E-03 |
|  |  |  | Weighted median | 58 | 0.27 | 0.08 | 1.25E-03 |
|  |  |  | Inverse variance weighted | 58 | 0.26 | 0.06 | 1.77E-05 |
| Lipoprotein subclasses | L_HDL_FC | 112.35 | MR Egger | 62 | -0.21 | 0.11 | 6.91E-02 |
|  |  |  | Weighted median | 62 | -0.22 | 0.09 | 1.83E-02 |
|  |  |  | Inverse variance weighted | 62 | -0.23 | 0.07 | 8.77E-04 |
| Lipoprotein subclasses | S_HDL_TG | 177.77 | Weighted median | 45 | 0.26 | 0.08 | 7.72E-04 |
|  |  |  | Inverse variance weighted | 45 | 0.29 | 0.06 | 2.78E-07 |
|  |  |  | MR Egger | 45 | 0.19 | 0.09 | 4.76E-02 |
| Relative lipoprotein lipid concentrations | M_HDL_TG_pct | 183.68 | Inverse variance weighted | 48 | 0.24 | 0.06 | 3.03E-05 |
|  |  |  | Weighted median | 48 | 0.21 | 0.08 | 5.07E-03 |
|  |  |  | MR Egger | 48 | 0.21 | 0.09 | 2.51E-02 |
| Lipoprotein particle sizes | VLDL_size | 136.74 | MR Egger | 49 | 0.11 | 0.10 | 2.80E-01 |
|  |  |  | Inverse variance weighted | 49 | 0.26 | 0.07 | 6.60E-05 |
|  |  |  | Weighted median | 49 | 0.29 | 0.09 | 1.57E-03 |

Metabolic biomarkers: the biomarkers used as exposures.

Metabolic group: the NMR biomarker subgroups.

F-Statistics: the F-statistics used to assess instrument strength. An F-statistic exceeding 10 indicates that substantial weak instrument bias is unlikely, with higher values corresponding to a lower bias risk.

Methods: the model used in MR causal estimation.

Beta: estimated coefficient indicating the causal effect of NMR metabolic biomarkers on CAC.

SE: standard error of Beta.

P-value: the statistical significance of a causal effect between NMR metabolic biomarkers and CAC.

**Supplementary Table 11: The sensitivity analysis of two-sample Mendelian randomization.**

| **Metabolic biomarkers** | **Direction test** | | | |  | **Pleiotropy Test** | | |  | **Heterogeneity Test** | | | |
| --- | --- | --- | --- | --- | --- | --- | --- | --- | --- | --- | --- | --- | --- |
|  | **SNP_r2.exposure** | **SNP_r2.outcome** | **Direction** | **P-value** |  | **Intercept** | **SE** | **P-value** |  | **Method** | **Q** | **Q_df** | **Q_pval** |
| Omega_6_pct | 0.04 | 2.37E-03 | TRUE | 2.20E-103 |  | -2.64E-03 | 0.01 | 0.71 |  | MR Egger | 33.18 | 35 | 0.56 |
|  |  |  |  |  |  |  |  |  |  | Inverse variance weighted | 33.32 | 36 | 0.60 |
| PUFA_by_MUFA | 0.04 | 2.38E-03 | TRUE | 6.56E-93 |  | -3.93E-03 | 0.01 | 0.61 |  | MR Egger | 32.88 | 34 | 0.52 |
|  |  |  |  |  |  |  |  |  |  | Inverse variance weighted | 33.14 | 35 | 0.56 |
| PUFA_pct | 0.03 | 2.27E-03 | TRUE | 4.06E-82 |  | -0.02 | 0.01 | 0.05 |  | MR Egger | 29.47 | 31 | 0.54 |
|  |  |  |  |  |  |  |  |  |  | Inverse variance weighted | 33.52 | 32 | 0.39 |
| L_HDL_CE_pct | 0.06 | 3.44E-03 | TRUE | 1.51E-168 |  | -4.21E-04 | 0.01 | 0.94 |  | MR Egger | 61.86 | 53 | 0.19 |
|  |  |  |  |  |  |  |  |  |  | Inverse variance weighted | 61.86 | 54 | 0.22 |
| L_HDL_C_pct | 0.06 | 3.40E-03 | TRUE | 2.89E-162 |  | -1.86E-03 | 0.01 | 0.72 |  | MR Egger | 54.62 | 55 | 0.49 |
|  |  |  |  |  |  |  |  |  |  | Inverse variance weighted | 54.75 | 56 | 0.52 |
| M_LDL_FC_pct | 0.06 | 3.12E-03 | TRUE | 1.53E-168 |  | -2.03E-04 | 0.01 | 0.97 |  | MR Egger | 54.74 | 45 | 0.15 |
|  |  |  |  |  |  |  |  |  |  | Inverse variance weighted | 54.74 | 46 | 0.18 |
| M_HDL_CE_pct | 0.06 | 3.11E-03 | TRUE | 2.50E-178 |  | -2.54E-03 | 0.01 | 0.67 |  | MR Egger | 47.88 | 46 | 0.40 |
|  |  |  |  |  |  |  |  |  |  | Inverse variance weighted | 48.07 | 47 | 0.43 |
| M_HDL_C_pct | 0.06 | 3.54E-03 | TRUE | 4.43E-180 |  | 7.74E-04 | 0.01 | 0.90 |  | MR Egger | 65.26 | 51 | 0.09 |
|  |  |  |  |  |  |  |  |  |  | Inverse variance weighted | 65.28 | 52 | 0.10 |
| L_HDL_CE | 0.08 | 4.05E-03 | TRUE | 3.26E-219 |  | -2.59E-03 | 0.01 | 0.61 |  | MR Egger | 85.04 | 69 | 0.09 |
|  |  |  |  |  |  |  |  |  |  | Inverse variance weighted | 85.36 | 70 | 0.10 |
| GlycA | 0.03 | 2.46E-03 | TRUE | 2.92E-73 |  | -0.02 | 0.01 | 0.09 |  | MR Egger | 39.94 | 33 | 0.19 |
|  |  |  |  |  |  |  |  |  |  | Inverse variance weighted | 43.71 | 34 | 0.12 |
| L_HDL_C | 0.07 | 3.83E-03 | TRUE | 1.14E-212 |  | -3.47E-03 | 0.01 | 0.50 |  | MR Egger | 79.89 | 66 | 0.12 |
|  |  |  |  |  |  |  |  |  |  | Inverse variance weighted | 80.45 | 67 | 0.13 |
| L_HDL_TG_pct | 0.07 | 3.69E-03 | TRUE | 8.16E-207 |  | 0.01 | 0.01 | 0.14 |  | MR Egger | 50.53 | 48 | 0.37 |
|  |  |  |  |  |  |  |  |  |  | Inverse variance weighted | 52.94 | 49 | 0.32 |
| TG_by_PG | 0.07 | 3.43E-03 | TRUE | 3.94E-193 |  | -2.54E-03 | 0.01 | 0.63 |  | MR Egger | 59.21 | 56 | 0.36 |
|  |  |  |  |  |  |  |  |  |  | Inverse variance weighted | 59.46 | 57 | 0.39 |
| L_HDL_FC | 0.06 | 3.77E-03 | TRUE | 4.27E-160 |  | -1.38E-03 | 0.01 | 0.82 |  | MR Egger | 78.76 | 60 | 0.05 |
|  |  |  |  |  |  |  |  |  |  | Inverse variance weighted | 78.84 | 61 | 0.06 |
| S_HDL_TG | 0.07 | 3.13E-03 | TRUE | 4.68E-201 |  | 0.01 | 0.01 | 0.16 |  | MR Egger | 28.85 | 43 | 0.95 |
|  |  |  |  |  |  |  |  |  |  | Inverse variance weighted | 30.86 | 44 | 0.93 |
| M_HDL_TG_pct | 0.07 | 3.29E-03 | TRUE | 2.47E-198 |  | 2.74E-03 | 0.01 | 0.66 |  | MR Egger | 54.25 | 46 | 0.19 |
|  |  |  |  |  |  |  |  |  |  | Inverse variance weighted | 54.48 | 47 | 0.21 |
| VLDL_size | 0.07 | 3.24E-03 | TRUE | 1.01E-189 |  | 0.01 | 0.01 | 0.08 |  | MR Egger | 50.25 | 47 | 0.35 |
|  |  |  |  |  |  |  |  |  |  | Inverse variance weighted | 53.69 | 48 | 0.27 |

Direction test: Steiger directionality test. SNP_r2.exposure: NMR metabolic biomarkers variance explained by the instruments. SNP_r2.outcome: CAC variance explained by the instruments. Direction: MR Steiger's directionality test to imply the true causal association’s direction. A P-value less than 0.05 was considered statistically significant evidence of correct causal direction.

Pleiotropy Test: Horizontal pleiotropy test from MR-Egger. Intercept: the intercept value from Egger regression. SE: Standard error. P-value: Strength of evidence against the null hypothesis of no pleiotropic effect.

Heterogeneity Test: Heterogeneity test using Cochran’s Q statistics; Q: Cochran’s Q statistic. Q_df: Cochran’s Q statistic degree of freedom. Q_pval: associated p-value with Cochran’s Q statistic.
